# Ion mobility mass spectrometry unveils conformational effects of drug lead EPI‐001 on the intrinsically disordered *N*‐terminal domain of the androgen receptor

**DOI:** 10.1002/pro.5254

**Published:** 2024-12-12

**Authors:** Ikhlas M. M. Ahmed, Adam Rofe, Martyn C. Henry, Eric West, Craig Jamieson, Iain J. McEwan, Rebecca Beveridge

**Affiliations:** ^1^ Department of Pure and Applied Chemistry University of Strathclyde Glasgow UK; ^2^ Institute of Medical Sciences, School of Medicine, Medical Sciences and Nutrition University of Aberdeen Aberdeen UK

**Keywords:** androgen receptor, EPI‐001, intrinsically disordered proteins, ion mobility mass spectrometry, native mass spectrometry, prostate cancer, protein–drug interactions

## Abstract

Intrinsically disordered proteins (IDPs) are important drug targets as they are key actors within cell signaling networks. However, the conformational plasticity of IDPs renders them challenging to characterize, which is a bottleneck in developing small molecule drugs that bind to IDPs and modulate their behavior. In relation to this, ion mobility mass spectrometry (IM‐MS) is a useful tool to investigate IDPs, as it can reveal their conformational preferences. It can also offer important insights in drug discovery, as it can measure binding stoichiometry and unveil conformational shifts of IDPs exerted by the binding of small drug‐like molecules. Herein, we have used IM‐MS to investigate the effect of drug lead EPI‐001 on the disordered *N*‐terminal domain of the androgen receptor (AR‐NTD). Despite structural heterogeneity rendering the NTD a challenging region of the protein to drug, this domain harbors most, if not all, of the transcriptional activity. We quantify the stoichiometry of EPI‐001 binding to various constructs corresponding to functional domains of AR‐NTD and show that it binds to separate constructs containing transactivation unit (TAU)‐1 and TAU‐5, respectively, and that 1–2 molecules bind to a larger construct containing both sequences. We also identify a conformational shift upon EPI‐001 binding to the TAU‐5, and to a much lesser extent with TAU‐1 containing constructs. This work provides novel insight on the interactions of EPI‐001 with the AR‐NTD, and the structural alterations that it exerts, and positions IM‐MS as an informative tool that will enhance the tractability of IDPs, potentially leading to better therapies.

## 1 INTRODUCTION

Intrinsically disordered proteins (IDPs) and intrinsically disordered regions (IDRs) are highly prevalent in biology (Uversky 2019; Wright and Dyson 2015), with bioinformatic studies indicating that around 25%–30% of eukaryotic proteins are disordered (Leuenberger et al. 2017). Due to their important role in many biological functions and being pivotal to protein interaction networks (Bondos et al. 2022), IDPs are extensively associated with human diseases including cancer, neurodegenerative diseases, and amyloidosis (Uversky et al. 2008). While this makes IDPs attractive drug targets, they represent challenging targets for inhibition by small molecules due to their dynamic and heterogeneous conformational preferences. Current strategies for targeting IDPs include inhibiting their interactions with binding partners, blocking their aggregation, and inhibiting liquid–liquid phase separation (Saurabh et al. 2023; Xie et al. 2022). A further possibility is to induce changes in the dynamic propensity of the IDP or IDR with a small molecule, thereby affecting its conformational distribution, and hence its function in the cell (Ban et al. 2017). In order to detect these binding events, techniques are required to measure small changes to the conformations of IDPs, which would expedite their exploitation as drug targets with novel therapeutics.

**FIGURE 1:**
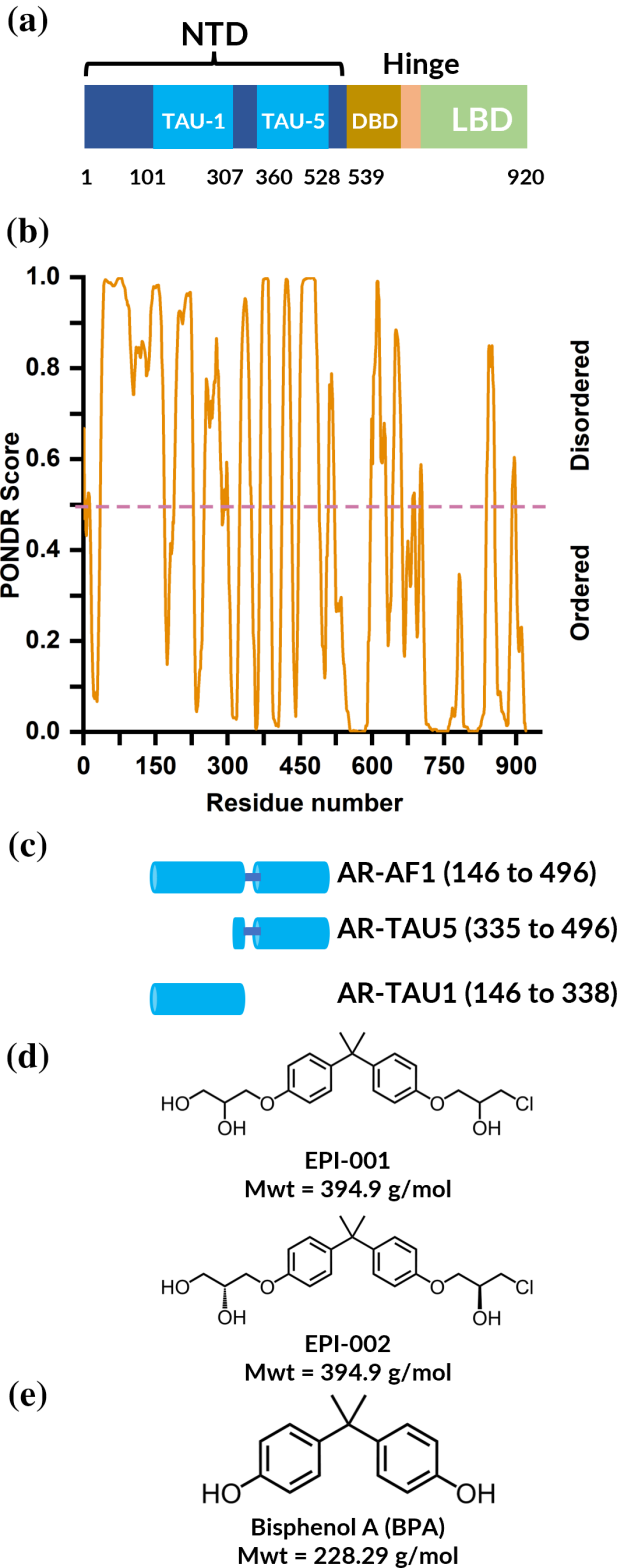
Domains of AR and their predicted intrinsic disorder. A schematic representation of AR domains, where TAU‐1 and TAU‐5 are predicted binding sites of EPI‐001 (a) (Monaghan and McEwan 2016). Predictions of disordered domains using PONDR where a score of 0.5 and above (shown by the dashed line) indicate disorder (b). A schematic depicting AR‐NTD constructs utilized in this study (c). The chemical structure of inhibitors EPI‐001 and EPI‐002 (d), and Bisphenol A (BPA) (e) which was used as a control molecule which does not bind to AR‐NTD.

One protein which contains an IDR and would benefit from additional biophysical measurement capabilities is the androgen receptor (AR) (Monaghan and McEwan 2016). This system plays a key role in the pathogenesis of prostate cancer (PC) which is associated with the fifth largest number of cancer deaths globally (Sung et al. 2021). The AR consists of an *N‐*terminal domain (NTD), a DNA‐binding domain (DBD), and a *C*‐terminal ligand binding domain (LBD) (Figure 1a). Both the LBD and the DBD have been structurally characterized via x‐ray crystallography (Nadal et al. 2017; Shaffer et al. 2004). The DBD is composed of two perpendicular α‐helices packed in perpendicular fashion, one of which inserts directly into the major groove of the DNA and makes key interactions with the DNA (Shaffer et al. 2004). The LBD was found to dimerise upon binding receptor agonists, which is an essential step in its proper functioning (Nadal et al. 2017). The *N*‐terminal domain is predicted to be intrinsically disordered by the algorithm predictor of natural disordered regions (PONDR) (Peng et al. 2005; Peng et al. 2006; Romero et al. 2001) (Figure 1b), and as determined experimentally by both circular dichroism, Fourier transform infrared spectroscopy and nuclear magnetic resonance (NMR) spectroscopy (De Mol et al. 2016; Kumar et al. 2004; Reid et al. 2002). Despite structural heterogeneity rendering the NTD a challenging region of the protein to identify a drug‐like ligand for, this domain contains most, if not all, of the transcriptional activity. Specifically, transactivation unit 1 (TAU‐1), and transactivation unit 5 (TAU‐5), shown in light blue in Figure 1a, are known functional domains that form transient α‐helices (Callewaert et al. 2006; De Mol et al. 2016). All current therapies that target AR involve reducing the level of circulating androgens by androgen ablation, and anti‐androgens against the LBD (Sadar 2011). Having stated this, the advanced form of the disease fails to respond to these strategies. This is the case in castration‐resistant prostate cancer (CRPC) (Antonarakis et al. 2016; Fujita and Nonomura 2019; Sadar 2011) where splice variants lacking the LBD, or missense mutations in the LBD, lead to loss of efficacy (Hay and McEwan 2012). In this case, only inhibition of the receptor through targeting other domains such as the NTD will prevent the AR from functioning in both the presence and absence of androgen.

Small molecules that bind the AR‐NTD have been identified from screening compound libraries (Andersen et al. 2010; Monaghan et al. 2022; Myung et al. 2013; Riley et al. 2023; Sadar 2020). The compounds include EPI‐001, EPI‐002 (Figure 1d), and related assets that are derived from a common Bisphenol‐A (BPA) scaffold (Figure 1e). EPI‐002 is a single diastereoisomer of EPI‐001. These EPI molecules have been shown to have an inhibitory effect on the transcriptional activation of AR‐NTD irrespective of the absence or presence of androgens and the LBD (Andersen et al. 2010; Banuelos et al. 2020; De Mol et al. 2016; Myung et al. 2013; Sadar 2011; Zhu et al. 2022). It has been proposed that a non‐covalent interaction between EPI compounds and AR‐NTD is followed by a covalent modification of the protein by the chlorohydrin group and that the initial non‐covalent interaction localizes the reactive ligands to specific cysteines of the protein (De Mol et al. 2016; Myung et al. 2013; Zhu et al. 2022). As such, a more holistic understanding of the initial non‐covalent binding event will be beneficial in developing further molecules with increased affinity for the AR‐NTD, leading to potentially differentiated mechanisms of action. Indeed, the interaction between EPI‐001 and AF1 has been extensively examined via biophysical techniques including fluorescence spectroscopy (Andersen et al. 2010; Myung et al. 2013), NMR (De Mol et al. 2016; Myung et al. 2013), gel electrophoresis (Brand et al. 2015; De Mol et al. 2016), molecular dynamics (MD) simulations (Zhu et al. 2022), and molecular docking (Sheikhhassani et al. 2022; Tran et al. 2020). In NMR studies, the interaction of EPI‐001 with the AR‐NTD has been shown to involve three regions with some degree of helical propensity (R1: 341–371, R2: 391–414, R3: 426–446) in transactivation unit 5 (TAU‐5) (De Mol et al. 2016) which is a functional domain of the AR‐NTD. Subsequently, MD simulations of a 56‐residue TAU‐5 fragment containing the R2 and R3 helices suggested that EPI‐002 and a structurally related analogue EPI‐7170 bind at the interface between the helices and induce the formation of partially folded collapsed helical states (Sheikhhassani et al. 2022; Zhu et al. 2022).

Native mass spectrometry (Barth and Schmidt 2020; Beveridge et al. 2014; Beveridge et al. 2016; Beveridge and Calabrese 2021; Hernández and Robinson 2007; Stuchfield and Barran 2018) (nMS) and the hybrid method ion mobility mass spectrometry (IM‐MS) are attractive techniques for analyzing IDPs (Robb et al. 2023), and have the potential to overcome difficulties encountered in addressing their tractability as therapeutic targets. With the use of nanoelectrospray ionization (nESI), which is known as a soft ionization technique, non‐covalent interactions can be maintained upon ionization which allows proteins and protein complexes to retain aspects of their native conformations as they are transferred into the gas phase (Beveridge and Calabrese 2021). nMS can therefore deduce stoichiometric information regarding protein complexes or protein–drug interactions according to their intact mass, and the number of charge states reveals the range of conformations adopted by a protein. Compact conformations have a limited surface area available for protonation, resulting in a relatively low charge compared to extended conformations that have many available protonation sites and therefore carry a high number of charges. Therefore, proteins with a compact structure have a narrow charge state range (Δ*z* ≤ 6) whereas IDPs have a wider charge state distribution that is reflective of their broader conformational range (Beveridge et al. 2014).

In IM‐MS analysis (Christofi and Barran 2023; Dodds and Baker 2019; Eyers et al. 2018; Ruotolo et al. 2008), additional information can be obtained regarding the size distribution of individual charge states of a protein or protein complex. This is due to separation based on the rotationally averaged collisional cross section (CCS) of the ions within an IM drift region, as larger conformations will travel more slowly due to increased interactions with an inert buffer gas. The experiment yields arrival time distributions (ATDs) which correspond to conformational distributions of a protein and allow comparison between conformational states of IDPs, for example, to observe how they are affected by drug or ligand binding. While CCSs, in units of Å^2^ or nm^2^, can be directly calculated from arrival time with the use of drift tube IM‐MS apparatus, the use of traveling wave IM‐MS, as used in this study, requires measuring appropriate calibrant proteins with known CCS values. The low sample requirement of IM‐MS, along with rapid data acquisition and low data processing positions it as an attractive tool in protein structure research. As stated above, therapeutic targeting of IDPs is currently challenging due to their incompatibility with many structural techniques, however, the potential of IM‐MS to reveal discrete conformations of IDPs under different conditions is particularly advantageous (Dickinson et al. 2015).

In this paper, we have used IM‐MS to measure the dynamic behavior of multiple constructs derived from the AR‐NTD:AR‐AF1 (containing TAU‐1 and TAU‐5; amino acids 146–496), AR‐TAU1 (amino acids 146–338), and AR‐TAU5 (amino acids 335–496) (Figure 1a,c). We have interrogated the interactions of these proteins with the known inhibitor EPI‐001 (Figure 1d), detailing unexpected stoichiometries of the protein–drug interactions, and revealing conformational alterations induced by its binding. Our studies on the interaction between AR‐NTD and EPI‐001 demonstrate the utility of IM‐MS as a tool for characterizing intrinsically disordered protein targets, and thus enhancing their tractability.

## 2 RESULTS

### 2.1 nMS quantifies the stoichiometry of AR‐AF1:EPI‐001 interactions

**FIGURE 2:**
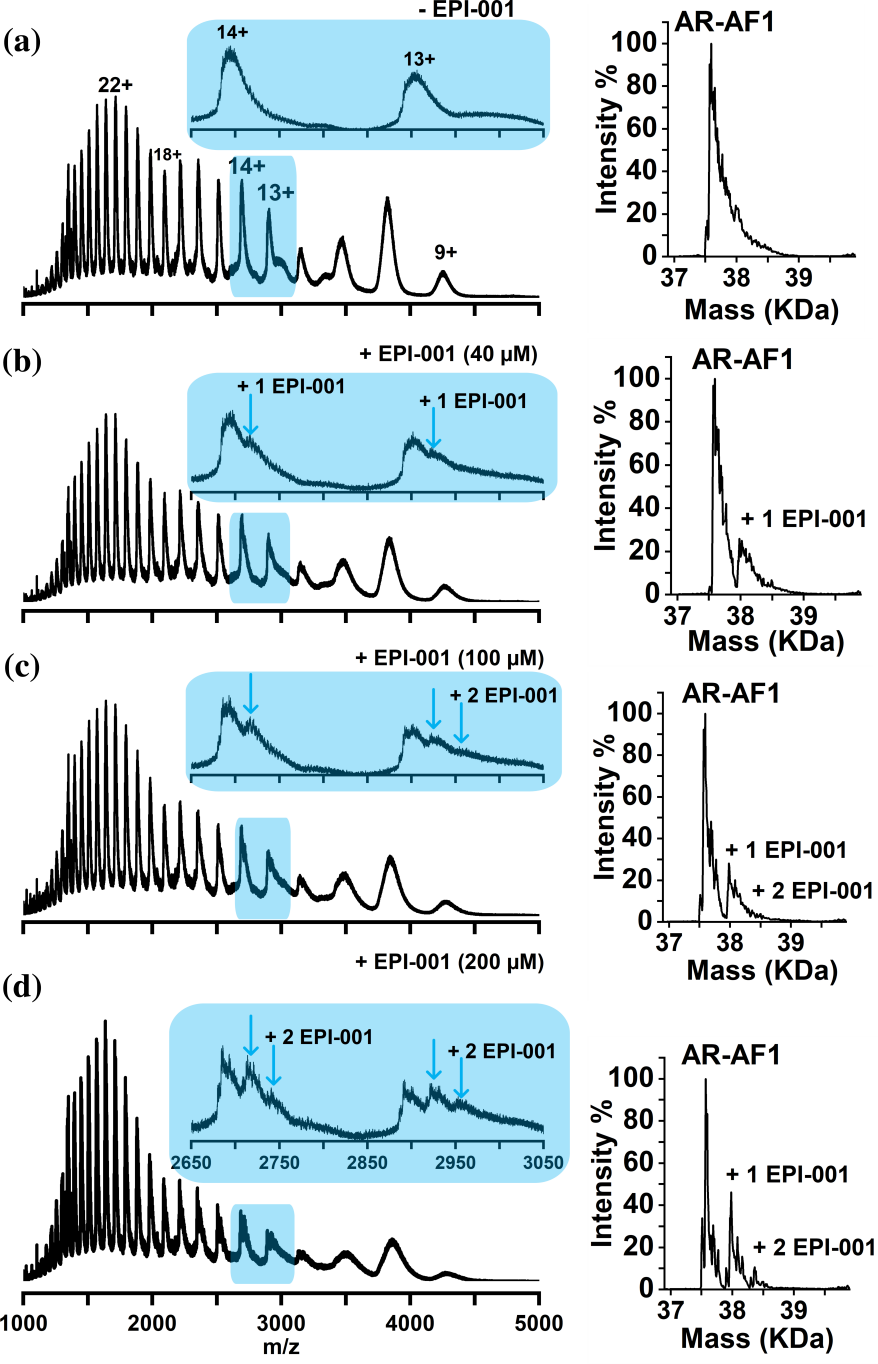
nMS of AR‐AF1 (4 μM) analyzed from AmAc (10 mM) pH 6.8, 1% DMSO in the absence (a) and presence of EPI‐001 at 40 μM (b), 100 μM (c), and 200 μM (d). Each spectrum is enlarged in the region of 2650 to 3050 *m*/*z*, where one (blue arrow) and two molecules (second blue arrow) of EPI‐001 binding to charge states 13+ and 14 is shown. Deconvoluted spectra of peaks in the *m*/*z* range of 2000–5000 are shown on the right of each spectrum. Molecular weight values of AR‐AF1 are shown in Table S1.

To investigate the effect of EPI‐001 on AR‐NTD, we first sought to characterize AR‐AF1 in the absence and presence of EPI‐001 at increasing concentrations via nMS (Figure 2). In the absence of EPI‐001, AR‐AF1 exists in charge states 9+ to 34+ (Figure 2a), which is a wide charge state distribution typical of an IDP (Beveridge et al. 2014). The trimodal distribution of charge states, with maxima at 10+, 14+, and 22+, suggests conformational families that are compact, intermediate, and extended, respectively (Beveridge et al. 2019).

To measure the extent of binding of EPI‐001 to AR‐AF1, EPI‐001 was added to the starting solution of AR‐AF1 at concentrations of 40, 100, and 200 μM (Figure 2b–d, respectively). With starting concentrations of 40 and 100 μM, EPI‐001 is observed to bind to charge states 9+ to 15+, which correspond to compact and intermediate conformational families. At a concentration of 200 μM, EPI‐001 is also observed binding to charge states 16+ to 19+, which are slightly more elongated conformations. Charge states 9+ and 10+ are broad due to incomplete desolvation during ionization and/or retention of salt, rendering exact binding stoichiometry difficult to determine. However, stoichiometric information about drug binding can be deduced from charge states 13+ and 14+ as shown in the insets in Figure 2b–d. At 40 μM EPI‐001, the molecule can be seen bound to the 13+ and 14+ charge states in a 1:1 stoichiometry (Figure 2b). At 100 μM EPI‐001 the signal intensity for the 1:1 complex is similar to 40 μM (Figure 2c), and a peak corresponding to the binding of a second molecule of EPI‐001 can also be observed upon close inspection in the 13+ charge state. At 200 μM EPI‐001 the binding of this second molecule is observed to a much greater extent, to both 13+ and 14+ charge states, demonstrating a 1:2 protein:drug stoichiometry (Figure 2d).

The deconvoluted spectra, shown to the right‐hand side (RHS) of each spectrum, represent the mass and intensity of each species as calculated from the individual charge states and allow a more quantitative assessment of the data. When EPI‐001 is present at 40 μM in the starting solution, the relative intensity of the peak corresponding to the 1:1 AR‐AF1:EPI‐001 complex is 26%. At 100 μM the relative signal intensity for the 1:1 and 1:2 peaks are 28% and 4%, respectively, and these values rise to 46% and 9%, respectively, for the sample containing 200 μM EPI‐001.

To address the possibility of non‐specific binding, AR‐AF1 was analyzed in the presence of BPA, which is a synthetic precursor to EPI‐001 lacking key functional groups that are critical for binding (Andersen et al. 2010). BPA was not observed to bind to AR‐AF1 when present in the starting solution at a concentration of 200 μM (Figure S1, Supporting Information). AR‐AF1 was also analyzed in the presence of EPI‐001 (200 μM) under denaturing conditions (pH 2) to disrupt any transient helices that are thought to be required for binding (De Mol et al. 2016). EPI‐001 binding is only observed in trace amounts under these conditions (Figure S2). To determine whether analysis from a higher salt concentration would affect the binding, nMS was also performed with a starting solution of 55 mM AmAc (Figure S3). Binding of EPI‐001 in a 1:1 stoichiometry is observed at ligand concentrations of 40 and 100 μM, similar to analysis from 10 mM AmAc, and the binding of a second EPI‐001 molecule can also be observed at 200 μM. This higher salt concentration therefore has little effect on the binding of EPI‐001 to AR‐AF1. Molecular weight values of AR‐AF1 are shown in Table S1.

### 2.2 AR‐TAU1 binds EPI‐001 to a higher extent than AR‐TAU5

**FIGURE 3:**
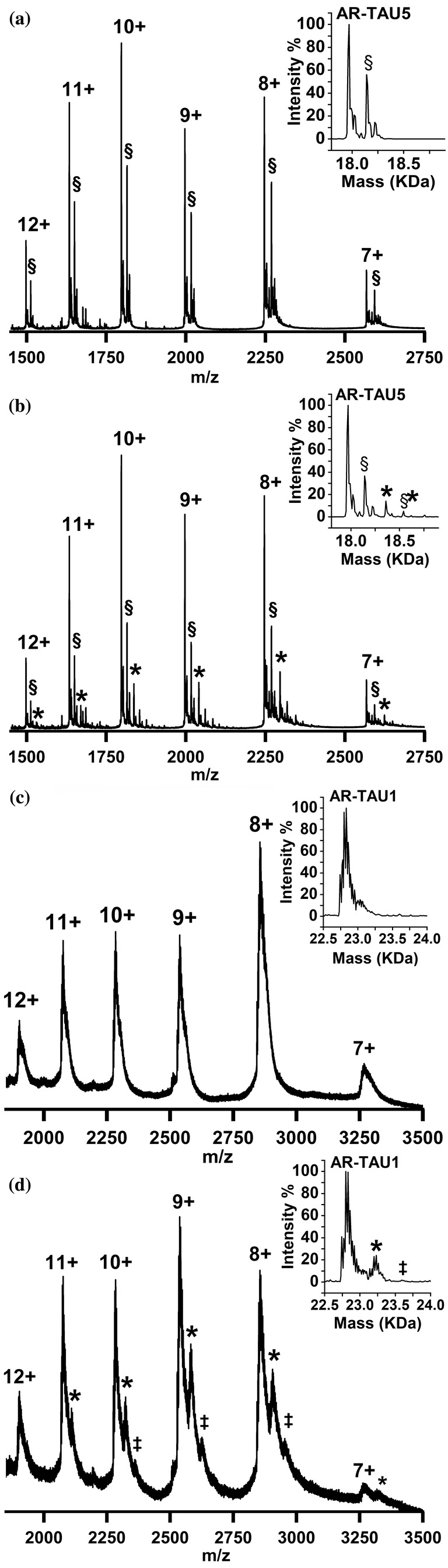
nMS of AR‐TAU5 (a, b) and AR‐TAU1 (c, d) in the absence (a, c) and presence (b, d) of EPI‐001. In sections b and d, samples were incubated in Tris buffer + EPI‐001 (200 μM) for 24 h before buffer exchange into 55 mM AmAc. Complexation with EPI‐001 is indicated by symbols * for 1:1, ‡ for 1:2 protein to drug complex, and § for adducts. Deconvoluted spectra are shown in the insets. Molecular weight values of AR‐TAU1 and AR‐TAU5 are shown in Table S1. Full spectra can be found in Figure S4.

EPI‐001 is hypothesized to target TAU‐5 of the AR‐NTD (De Mol et al. 2016). We therefore sought to characterize its binding to AR‐TAU5 which is a shorter construct that contains the regions of helical propensity of TAU‐5 (residues 390–410, 335–365), and those residues vital for enabling protein–protein interactions (433–437) which are thought to be collectively important for binding EPI‐001 (De Mol et al. 2016). For initial analysis, AR‐TAU5 was ionized from a solution of 55 mM AmAc and was found to display a wide charge state distribution from 7+ to 18+ (Figures 3a and S4a), typical for a disordered protein. A second peak at roughly 50% relative intensity to the unbound protein is also observed for each charge state (labeled §), which corresponds to a mass adduct of 174 Da, and is likely a plasticizer molecule from plasticware used in the lab. In the deconvoluted spectrum (inset), the relative intensity of this peak is ~60% relative to the unbound protein. This adduct is also found on other constructs but is less apparent due to increased width of the peaks.

To investigate the binding of EPI‐001 to AR‐TAU5, a new method of sample preparation was developed that involved incubating the protein and ligand (10 and 200 μM, respectively) in Tris buffer at 4°C for 24 h, before buffer‐exchanging the sample into AmAc (55 mM) via microdialysis immediately before nMS analysis. This method was initially developed to promote the covalent interaction between EPI‐001 and AR‐TAU5 that has been hypothesized to form. While the evidence surrounding the covalent attachment of EPI‐001 remains inconclusive, as described below, the method had the unexpected advantage of lower salt retention compared to analysis of the sample by the traditional method of performing buffer exchange and subsequent addition of the ligand. It also meant that any “free” ligand was removed during dialysis, therefore reducing the propensity of non‐specific protein–ligand complexes being induced during desolvation.

After 24‐h incubation of AR‐TAU5 with EPI‐001 in Tris buffer and subsequent buffer exchange into 55 mM AmAc, nMS reveals EPI‐001 binding to AR‐TAU5 in the 7+ to 12+ charge states, which is labeled as * in Figure 3b. Signal intensity corresponding to the AR‐TAU5:EPI‐001 complex is 14% relative to the unbound protein, as shown in the deconvoluted spectrum (inset). Sample preparation via the traditional method, in which AR‐TAU5 was first buffer exchanged into AmAc and EPI‐001 subsequently added to a final concentration of 100 μM and incubated for 10 min, yielded similar results but with broader peaks (Figure S5). Upon increasing the concentration of EPI‐001 to 200 μM, a slightly higher relative signal intensity of around 30% is observed (Figure S6).

A key question that we strove to answer during these experiments is whether the EPI‐001 molecule is covalently or non‐covalently bound to AR‐TAU5. To address this, we first considered the mass difference between the unbound AR‐TAU5 and the EPI‐001‐bound version which was calculated to be 394.9 Da, indicating that the chlorine atom is still attached to EPI‐001 and therefore suggesting that no covalent modification of the protein has occurred (see Figure S7). However, after denaturing this complex via its acidification to pH 2 (Figure S7b), the peak corresponding to the protein–ligand complex can still be observed, which suggests covalent attachment (Figure S7b). As an additional test, the 7+ charge state of the protein–ligand complex was isolated in an MS/MS experiment, to measure whether the EPI‐001 molecule could be easily dissociated from the protein upon collisional activation. Even without any additional collisional energy (CE), a sodiated adduct of EPI‐001 was dissociated from the complex, leaving behind the 7+ unbound protein ion (Figure S8). Upon increasing the CE to 10 V, the intensity of the peak corresponding to the unbound protein increases so that it is slightly higher than the intensity of the protein–ligand complex, and upon increasing the CE further to 15 V the EPI‐001 is completely dissociated from the protein. In contrast to the denaturing experiments, this MS/MS data suggests non‐covalent attachment of EPI‐001 to AR‐TAU5 as it is readily dissociated from the protein in the gas phase. We therefore remain uncertain as to whether EPI‐001 is covalently or non‐covalently bound to AR‐TAU5.

We next characterized the binding of EPI‐001 to AR‐TAU1, which also has regions of helical propensity including residues 185–200 and 230–240 (De Mol et al. 2016). This region is also thought to participate in the binding of EPI‐001, as studies have identified EPI‐001 binding to AR‐NTD lacking TAU‐5 (Brand et al. 2015). AR‐TAU1 also displays a wide charge state distribution from 7+ to 14+ (Figures 3c and S4c). Here, peaks are slightly broader than for AR‐TAU5, likely due to increased solvent and salt retention which often differs between proteins. Nevertheless, peaks are still narrow enough to unambiguously observe the binding stoichiometries of EPI‐001 to AR‐TAU1. Upon incubating AR‐TAU1 (10 μM) with EPI‐001 (200 μM) for 24 h in Tris buffer and subsequent buffer exchange into 55 mM AmAc, 1:1 complexes are observed in charge states 7+ to 11+ (Figure 3d). The signal intensity corresponding to the 1:1 complex ranges from 30% relative to the unbound protein in charge state 11+ to 50% in charge state 8+. Interestingly, a low amount of signal corresponding to a 1:2 protein to drug complex is observed in charge states 8+ to 10+ indicating the binding of two EPI‐001 molecules to one AR‐TAU1 molecule. Overall, in the deconvoluted spectrum, the signal intensity of the peak corresponding to the AR‐TAU1:EPI‐001 complex in the 1:1 stoichiometry is 24% relative to the unbound protein, and that in the 1:2 stoichiometry is shown to be 1%. However, it appears that the 1:2 complex is underrepresented in the deconvoluted spectrum, due to the width of the peaks which proves challenging for the software to interpret. Sample preparation via the progenitor method, in which AR‐TAU1 was first buffer exchanged into AmAc and EPI‐001 subsequently added to a final concentration of 100 μM and incubated for 10 min, resulted in a 1:1 complex in charge states 7+ to 13+ (Figure S5). Upon increasing the EPI‐001 concentration to 200 μM, 1:2 protein to drug complexes were also observed (Figure S6). Interactions between AR‐TAU1 and BPA were only observed to a very low extent (Figure S9), demonstrating specificity of the binding of EPI‐001. These data suggest that the AR‐TAU1 construct binds EPI‐001 with higher affinity than AR‐TAU5. This result is unexpected, as the majority of the prior literature suggests that TAU5 is the predominant binding site for EPI‐001 (De Mol et al. 2016; Zhu et al. 2022). Nevertheless, the binding to AR‐TAU1 is specific, as demonstrated by the low level of binding by BPA, and is therefore worthy of further investigation.

### 2.3 EPI‐001 has a greater effect on the conformational distribution of AR‐TAU5 than AR‐TAU1

**FIGURE 4:**
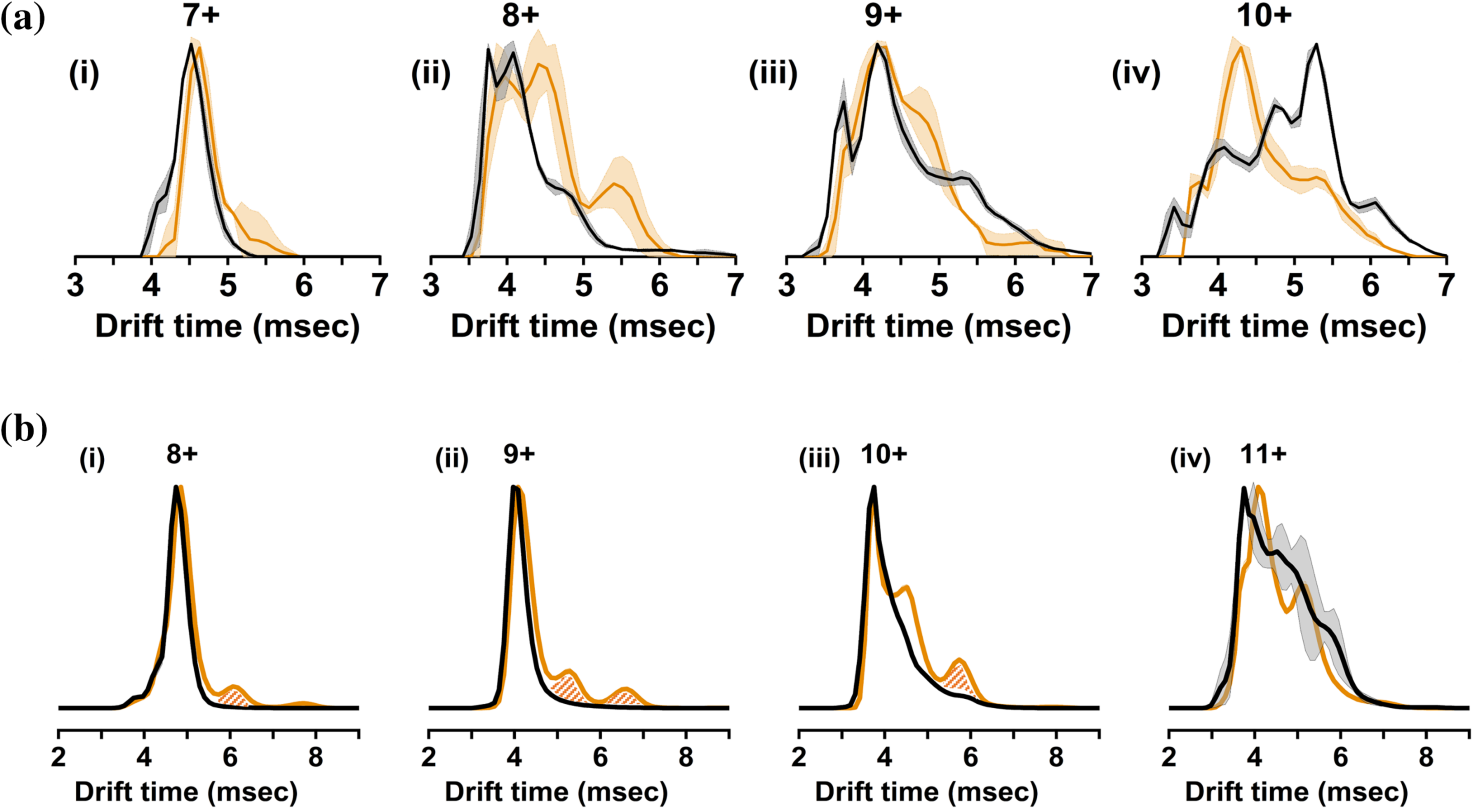
(a) IM traces of charge states 7+ (i), 8+ (ii), 9+ (iii), and 10+ (iv) of AR‐TAU5. (b) IM traces of charge states 8+ (i), 9+ (ii), 10+ (iii), and 11+ (iv) of AR‐TAU1. Black line: control (1% DMSO). Orange line: protein–drug complex at 1:1 stoichiometry. The solid lines correspond to the average of three repeats, and the light shaded area corresponds to standard deviation. Peaks filled with a hatched pattern in part b are due to signal interference and not conformational change.

As well as informing on the stoichiometry of the complexes formed via MS, our experiments can also reveal conformational changes exerted on the proteins by the binding of small molecules via IM‐MS. Figure 4a shows the ATDs of charge states 7+ to 10+ of AR‐TAU5 for the unbound (black line) and EPI‐001‐bound species (orange line), which represent the size distribution of AR‐TAU5 and the TAU5‐EPI‐001 complex, respectively. The ATD of the unbound species was obtained by combining over the corresponding charge state peak of the control sample, while the ATD of the protein–drug complex was determined by combining over the peak corresponding only to the 1:1 complex of the sample containing EPI‐001. Therefore, theoretically these mass‐selected ATDs are not affected by other species present in the mixture. In practice, when viewing the 2‐diminsional (2D) heatmaps corresponding to our IMMS data, some “smearing” of the peaks could be observed which resulted in a small amount of signal interference upon the analysis of AR‐TAU1. We therefore systematically crosschecked our ATDs with the 2D heatmaps (representative plots shown in Figures S10 and S12 for AR‐TAU5 and AR‐TAU1, respectively). Importantly this allowed us to discount any data features which arose due to interference, which are filled with a hatched pattern in Figure 4b.

The 7+ unbound species of AR‐TAU5 has a narrow monomodal ATD with an apex of 4.52 ms, representative of a single compact conformational family. The addition of EPI‐001 causes a small shift of the ATD to a slightly longer drift time (apex 4.63 ms) representative of a small increase in the size of the protein. Moreover, the RHS of the peak now extends to a higher arrival time of 5.84 ms compared to 5.29 ms for the unbound protein, indicating a slight elongation of the most extended species. The 8+ charge state also indicates elongation of AR‐TAU5 upon binding of EPI‐001, with increased intensity at higher drift times up to 6.00 ms. While EPI‐001 slightly changes the apex of the peak corresponding to the ATD of the 9+ charge state (4.19 ms unbound vs. 4.30 ms bound), it causes a higher intensity at 4.80 ms, and a decrease in intensity at the later arrival time of ~5.50 ms, suggesting an overall decrease in the conformational spread of this charge state. The 10+ charge state is clearly more compact when bound to EPI‐001, with an apex at 5.29 ms for the unbound and 4.30 ms for the complex. EPI‐001 is generally thought to be a racemic mixture of four enantiomers, with the primary biological inhibitory activity found in the stereoisomer EPI‐002 (2R, 20S) (Banuelos et al. 2020; Myung et al. 2013). Therefore, following chemical synthesis of the single stereoisomer (appendix I and II in Data S1), the interaction with AR‐TAU5 was examined via IM‐MS (Figure S13). The complex formed between AR‐TAU5 and EPI‐002 exists in charge states 6+ to 10+ with a similar intensity as observed for EPI‐001. Additionally, the binding of EPI‐002 and EPI‐001 have similar effects on the drift time of the protein.

IM‐MS experiments were also carried out to discern the changes in the conformations of AR‐TAU1 upon binding EPI‐001. Here, the conformational changes are much smaller than with AR‐TAU5. For the 8+, 9+, and 10+ charge states there is almost no discernible change in drift time for the apex of the peaks upon the binding of one or two EPI‐001 molecules (Figures 4b and S11), meaning that the majority of the protein molecules remain in the same conformation upon EPI‐001 binding. The low‐intensity signal at higher drift time in charge states 8+ and 9+ is solely due to signal interference (see Figure S12). However, for 10+, an additional feature at 4.52 ms corresponds to a new conformation that is slightly larger than the unbound species. The 11+ charge state shows the biggest change upon EPI‐001 binding, with one broad peak partially resolving into two conformational families. The binding of a second EPI‐001 molecule can be seen not to induce any conformational change in charge states 8+ and 9+, and the signal intensity for this species is too low to be interpreted in charge states 10+ and 11+ (Figure S12).

### 2.4 EPI‐001 binding to AR‐TAU5 results in the stabilization of a new gas phase conformation

**FIGURE 5:**
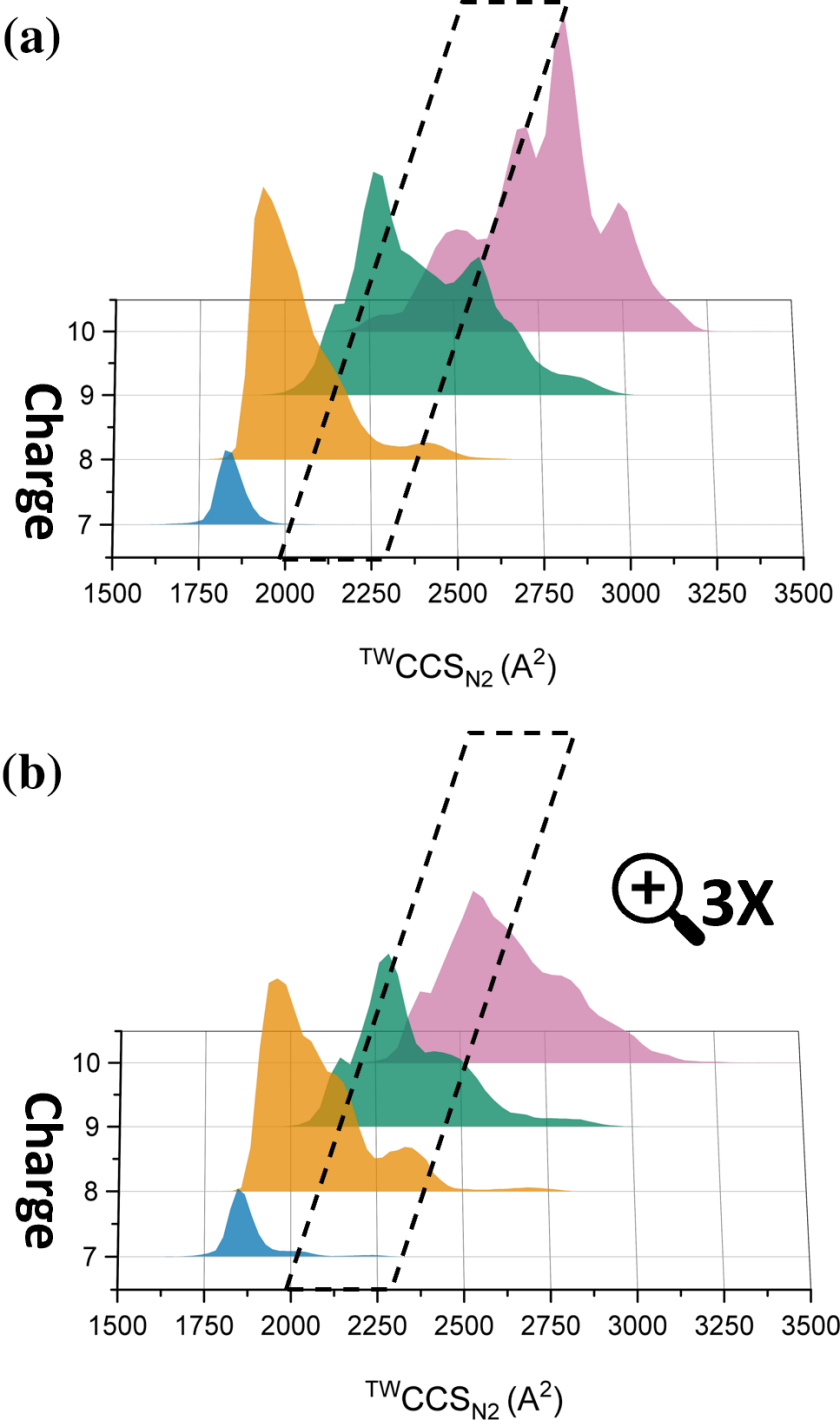
CCS distributions of (a) unbound AR‐TAU5, (b) EPI‐001‐bound AR‐TAU5 charge states. The black box highlights an intermediate conformational space, that is more highly populated by AR‐TAU5 in the presence of EPI‐001 via elongation of charge states 7+ and 8+ and compaction of 9+ and 10+. The height of each CCS distribution corresponds to the relative intensity of the corresponding peak in the mass spectrum shown in Figure 3b, with the bottom panel being magnified 3x due to the low signal intensity for the protein:molecule complex.

To better understand the effect of EPI‐001 binding to the conformation of AR‐TAU5, the IM data were calibrated against standard proteins of known sizes to obtain CCS distributions of AR‐TAU5, given in Å^2^ (Figure 5). These plots display the CCS distribution of each charge state in the absence and presence of EPI‐001 (Figure 5a,b, respectively). In the absence of EPI‐001 (Figure 5a), the CCS ranges are 1649–2099 Å^2^ (7+), 1774–2670 Å^2^ (8+), 1930–3021 Å^2^ (9+), and 2143–3258 Å^2^ (10+) and the apexes of the peaks are 1825 Å^2^ (7+), 1935 Å^2^ (8+), 2258 Å^2^ (9+), and 2825 Å^2^ (10+). Upon adding the EPI‐001, conformational changes to each charge state occur that increases the population inside the outlined area in Figure 5b. Specifically, the upper limit of the 7+ and 8+ charge states increase to 2274 and 2798 Å^2^, respectively. Two new conformational families of the 7+ charge state can be observed with maxima at 2024 and 2240 Å^2^, which are lowly populated but are inside the outlined area and absent when the protein is analyzed without EPI‐001. For the 8+ charge state, a shoulder on the right of the main peak becomes more intense and a conformational family at ~2300 Å^2^ appears; both of these features increase the conformational population inside the outlined area. The CCS distribution for the 9+ charge state becomes overall more compact, as the peak centered around 2600 Å^2^ is almost completely removed, showing that fewer molecules have this extended conformation upon binding EPI‐001. 10+ undergoes the highest degree of compaction upon EPI‐001 binding, resulting in an apex at a much lower CCS of 2537 Å^2^ that shifts most of the conformational population to within the outlined area. All these changes result in the increased population of the “intermediate” conformational space that is outlined in the black line, that is populated to a much lower extent in the control.

## 3 DISCUSSION

The first objective of this research was to quantify the binding of drug lead EPI‐001 to various constructs of the AR‐NTD to better understand the interactions leading to the associated pharmacology. Upon comparing EPI‐001 binding by the individual functional domains (AR‐TAU5 and AR‐TAU1) with the construct containing both (AR‐AF1), it was noticed that AR‐TAU5 binds in a 1:1 stoichiometry, AR‐TAU1 binds at 1:1 and 1:2, and AR‐AF1 also binds at 1:1 and 1:2. Interestingly, there is no 1:3 binding of AR‐AF1 with EPI‐001, despite the apparent possibility that would arise from the binding sites of AR‐TAU1 and AR‐TAU5 being present in a single construct. This is enhanced by the fact that the protein concentration of AR‐AF1 is 4 μM and that of AR‐TAU1 and AR‐TAU5 is 10 μM, meaning that the drug to protein ratio for AR‐AF1 is higher. This part of our study relates to work by De Mol et al. (2016), in which EPI‐001 binding to a similar construct to AR‐AF1 was analyzed by NMR. The authors primarily noted chemical shift perturbations to residues in TAU5, and to a much lesser extent in TAU1. It also relates to work by Sheikhhassani et al. (2022), who explored the structural plasticity of AR‐NTD via a combination of MD simulations and circuit topology analysis and located regions that are dynamic as well as those that are compact. The latter was further categorized into dynamic shell residues (225–354, 470–538) capable of engulfing the core residues (355–469). Both regions were found to be involved in the binding of EPI‐001, with shell residues rearranging to restrict further access of EPI‐001 to core residues.

The second objective was to delineate changes to the conformational distribution of the AR‐NTD upon binding of EPI‐001. As described above, IM‐MS measurements revealed that EPI‐001 binding to AR‐TAU5 causes a significant change in the arrival time distributions of the proteins, representative of conformational effects. AR‐TAU1, on the other hand, only undergoes a very minimal change in ATD, and hence conformation, upon EPI‐001 binding. We therefore conclude that while AR‐TAU1 has a higher binding affinity for EPI‐001 than AR‐TAU5, the molecule has a larger effect on the conformational distribution of TAU5 than TAU1. Delineating the effects of binding versus conformational change has only been possible through use IM‐MS. Therefore, the importance of targeting TAU1 or TAU5 in the development of AR inhibitors remains an open question, depending on whether molecule binding or impact on conformational rearrangement is the most relevant for a successful drug.

Finally, by converting the ATDs of AR‐TAU5 to CCS distributions (Figure 5), we have determined that binding of EPI‐001 stabilizes alternative gas phase conformations of the protein. For AR‐TAU5, this protein:ligand complex has an overall larger conformation in charge states 7+ and 8+ than the unbound protein, but an overall smaller conformation in charge states 9+ and 10+. We hypothesize that the protein:ligand complex therefore has increased helicity compared to the unbound protein, as increased helicity has been found to prevent IDPs from adopting compact gas phase structures (Harvey et al. 2012) and will also prevent occupancy of more elongated conformations. Moreover, increased helicity of AR‐TAU5 has also been shown via NMR spectroscopy (De Mol et al. 2016) as well as MD simulations (Zhu et al. 2022). We hypothesize that this gas‐phase conformation could be used as a fingerprint relating to the profile of a pharmacologically relevant ligand in future IM‐MS‐based screening methods aimed at identifying successor compounds to EPI‐001 and related analogues. A screening platform of this type would be advantageous, as it would reveal: (i) binding of a molecule, and (ii) the conformational preference of the protein which is associated with desired pharmacological activity, in a single experiment. Altering the conformational preferences of the AR‐NTD may reduce its ability to interact with transcriptional machinery, as has been demonstrated for the CREB binding protein (Andersen et al. 2010) and Rap74 (De Mol et al. 2018).

## 4 CONCLUSIONS

Despite the importance of the AR‐NTD as a drug target for novel treatments against advanced prostate cancer, little is known about its range of conformations, and how these are affected by the binding of small drug‐like molecules. Using IM‐MS, we have quantified the binding stoichiometry of a palette of protein constructs representing various regions of the AR‐NTD with the drug lead EPI‐001, and have elucidated the effect that this molecule can have on the conformational preferences of the protein.

In the current study, we have demonstrated the utility of IM‐MS in elucidating the binding of EPI‐001 to the AR‐NTD, and the conformational effect that it has on the protein. This new knowledge relating the binding mechanisms of this class of drug will be beneficial in the identification and development of a next generation of compounds with improved properties, potentially leading to enhanced treatments for prostate cancer. It is expected that the IM‐MS methods developed will be applicable to further intrinsically disordered drug targets, of which there are many. It also highlights the potential of IM‐MS to be a useful screening tool in the identification of novel drug leads against IDPs, which will be the focus of future work.

## 5 MATERIALS AND METHODS

### 5.1 Protein expression and purification

Recombinant AR‐NTD polypeptides, AR‐AF1, AR‐TAU1, and AR‐TAU5, were induced in bacteria (BLR cells) with 1 nM IPTG, for 2 h at 37°C. Expressed proteins were then purified by Ni‐affinity chromatography and eluted in Tris buffer (20 mM Tris, pH 7.9, 500 mM NaCl, 200 mM imidazole, and 5% glycerol). For all proteins, the concentration was determined by Bradford assay and purity confirmed by SDS‐polyacrylamide gel electrophoresis. The sequences of all proteins are given in Table S2.

### 5.2 Sample preparation for nMS and IM‐MS

Ammonium acetate solution (AmAc) was prepared at pH 6.8 from ultra‐pure water (18.2 MΩ·cm, Millipore) and analytical grade AmAc solid (Fisher Scientific). Small molecules (EPI‐001 from Tokyo Chemical Industry UK Ltd., BPA from Alfa Aesar, and EPI‐002) were weighed and dissolved in Dimethyl sulfoxide (DMSO, LC‐MS grade ≥99.7% purity, ThermoFisher) to make a stock solution of 20 mM which was diluted with water to a final concentration of 2 mM and 10% DMSO. This solution was used for protein‐small molecule studies at various final concentrations (40–200 μM) while maintaining a final 1% DMSO concentration.

For analysis in the absence of small molecules, protein samples were buffer exchanged into the relevant AmAc solutions (Table S3) using a 96‐well microdialysis plate (Thermo Fisher Scientific), the resulting protein concentration was determined with a NanoDrop spectrophotometer (Thermo Fisher Scientific) using the A280 method, and the samples were diluted to the relevant protein and AmAc concentrations (Table S3).

To measure small molecule binding via the traditional method (AR‐AF1, IM‐MS studies of AR‐TAU5, MS/MS studies pertaining to the AR‐TAU5:EPI‐001 interaction, control experiments shown in Data S1), the molecule was added to the protein post‐dialysis and the sample was incubated for 10 min on ice. For analysis of AR‐AF1 at pH 2, AmAc (10 mM) was adjusted to pH 2 using HCl, and this was used for dilution and incubation of the protein at pH 2 for 10 min before the addition of EPI‐001.

To measure EPI‐001 binding to AR‐TAU5 and AR‐TAU1 after extended incubation times in Tris buffer (MS studies of AR‐TAU5 and AR‐TAU1 in Figure 3, IMMS studies of AR‐TAU1 in Figure 4), the proteins (10 μM) were incubated in Tris buffer + EPI‐001 (200 μM) for 24 h at 4°C. Buffer composition for AR‐TAU5 was 10 mM Tris 250 mM NaCl 100 mM imidazole 2.5% v/v glycerol pH 7.9, and for AR‐TAU1 was 0.66 mM Tris 16.6 mM NaCl 6.66 mM imidazole 0.166% v/v glycerol pH 7.9. For analysis in the absence of EPI‐001, AR‐TAU1 was prepared in the same manner in the absence of the molecule but including 1% DMSO. The samples were subsequently buffer‐exchanged via microdialysis into 55 mM AmAc for MS analysis. For analysis of AR‐TAU5 + EPI‐001 at pH 2 after 24‐h incubation, the sample was adjusted to pH 2 after microdialysis using formic acid, and then incubated for 20 min before analysis. AR‐TAU5 in the absence of EPI‐001 was prepared via direct buffer exchange (microdialysis) into 55 mM AmAc. For determining rotationally averaged collisional cross section values (Ω), β‐lactoglobulin from bovine milk (Sigma), and Bovine serum albumin (Fisher) were dissolved in AmAc (100 mM), desalted using a Bio‐spin P6 column (BIO‐RAD) and diluted to protein concentrations of 5 μM for analysis.

### 5.3 Experimental parameters for IM‐MS and data processing

IM‐MS spectra were acquired on a Synapt G2‐Si mass spectrometer (Waters, Wilmslow, UK) with a nESI source operated in sensitivity mode. Solutions were ionized by applying a positive potential through a platinum wire (thickness 0.125 mm, Goodfellow) that was inserted into a thin‐walled glass capillary (inner diameter 0.78 mm, outer diameter 1 mm, 10 cm length, World Precision Instruments) that was pulled to a nESI tip in house with a Flaming/Brown micropipette puller (Sutter Instrument Co.) using the following settings: Pressure 500, Heat 475, Velocity between 28 and 34, Time 250. All values are arbitrary units. Non‐default instrument settings include capillary voltage 0.9–1.2 kV, sampling cone 30–40 V, source offset 40–60 V, source temperature 40°C, desolvation temperature 150°C, trap collision energy 5 V, and trap gas flow 3 mL/min. Data for AR‐AF1 analyzed from 55 mM AmAc are smoothed for clarity.

IM data were collected at traveling wave velocity and height of 400 m/s and 40 V, respectively. Helium and nitrogen (IMS) gas flows were 180 mL/min and 90 mL/min, respectively. The instrument was allowed to settle for 1 h prior to experiments. Data were acquired over *m*/*z* range 700–5000 except for bovine serum albumin which was acquired over *m*/*z* range 700–8000. For AR‐TAU1, a manual quad profile set to 2650 *m*/*z* was used. MS/MS experiments of the 7+ charge state of the 1:1 AR‐TAU5:EPI‐001 complex were carried out by isolating the species in the quadrupole (MS/MS mode) and increasing the collision energy in the trap region.

CCS calibration was performed using IMSCal (Richardson et al. 2021) with β‐lactoglobulin and bovine serum albumin (BSA) used as CCS calibrants (Bush et al. 2010). IM‐MS data were acquired and processed using MassLynx (V4.2, Waters). Data for AR‐TAU5 (Figure 4a) were additionally processed using driftscope (Waters, Wilmslow, UK). IM‐MS profiles were created in OriginPro 2022 (OriginLab Corporation) by extracting ATDs of selected charge states in the mass spectrum, then normalizing and averaging data collected from multiple emitters on different days. The standard deviation is displayed as the shaded area.

AR‐TAU1 and AR‐TAU5 data were deconvoluted using unidec (Marty et al. 2015), with non‐default data processing parameters as follows: *m*/*z* range 1000–5000, peak detection range 100 Da, peak detection threshold 0.01. Background subtraction was enabled. Equivalent parameters were used for AR‐AF1 except *m*/*z* range (2000–5000) and peak detection range (10 Da).

### 5.4 Synthesis of EPI‐002

An asymmetric route to EPI‐002 was utilized, details of which are provided in the SI (EPI‐002 synthesis), along with full characterization data (appendix I in Data S1). Figures were made using OriginPro 2022 and Inkscape (V1.2, Inkscape project).

## AUTHOR CONTRIBUTIONS

**Ikhlas M. M. Ahmed:** Writing – original draft; investigation; methodology; validation; visualization; formal analysis; data curation. **Adam Rofe:** Resources. **Martyn C. Henry:** Resources. **Eric West:** Resources. **Craig Jamieson:** Writing – review and editing; supervision. **Iain J. McEwan:** Writing – review and editing; supervision. **Rebecca Beveridge:** Supervision; conceptualization; funding acquisition; methodology; writing – review and editing; investigation.

## Supporting information


**Data S1.** Supporting Information.
