# Supplementary material for: Ion mobility mass spectrometry unveils conformational effects of drug lead EPI‐001 on the intrinsically disordered N‐terminal domain of the androgen receptor
Source: Protein Sci. 2024 Dec 12;34(1):e5254. doi: 10.1002/pro.5254 (PMC11635395; doi:10.1002/pro.5254)
Supplement: Supplementary file 1 — Data S1. Supporting Information. [file PRO-34-e5254-s001.docx]

**Supplementary Information**

**Ion mobility mass spectrometry unveils conformational effects of drug lead EPI-001 on the intrinsically disordered N-terminal domain of the Androgen Receptor**

Ikhlas M. M. Ahmed^1^, Adam Rofe^2^, Martyn C. Henry^1^, Eric West^2^ , Craig Jamieson^1^, Iain J. McEwan^2^, Rebecca Beveridge^1^*

^1^ Department of Pure and Applied Chemistry, University of Strathclyde, Glasgow, United Kingdom G1 1XL.

^2^ Institute of Medical Sciences, chool of Medicine, Medical Sciences and Nutrition, University of Aberdeen, Aberdeen, United Kingdom. AB25 2ZD.

* Correspondence: [rebecca.beveridge@strath.ac.uk](mailto:rebecca.beveridge@strath.ac.uk)

**
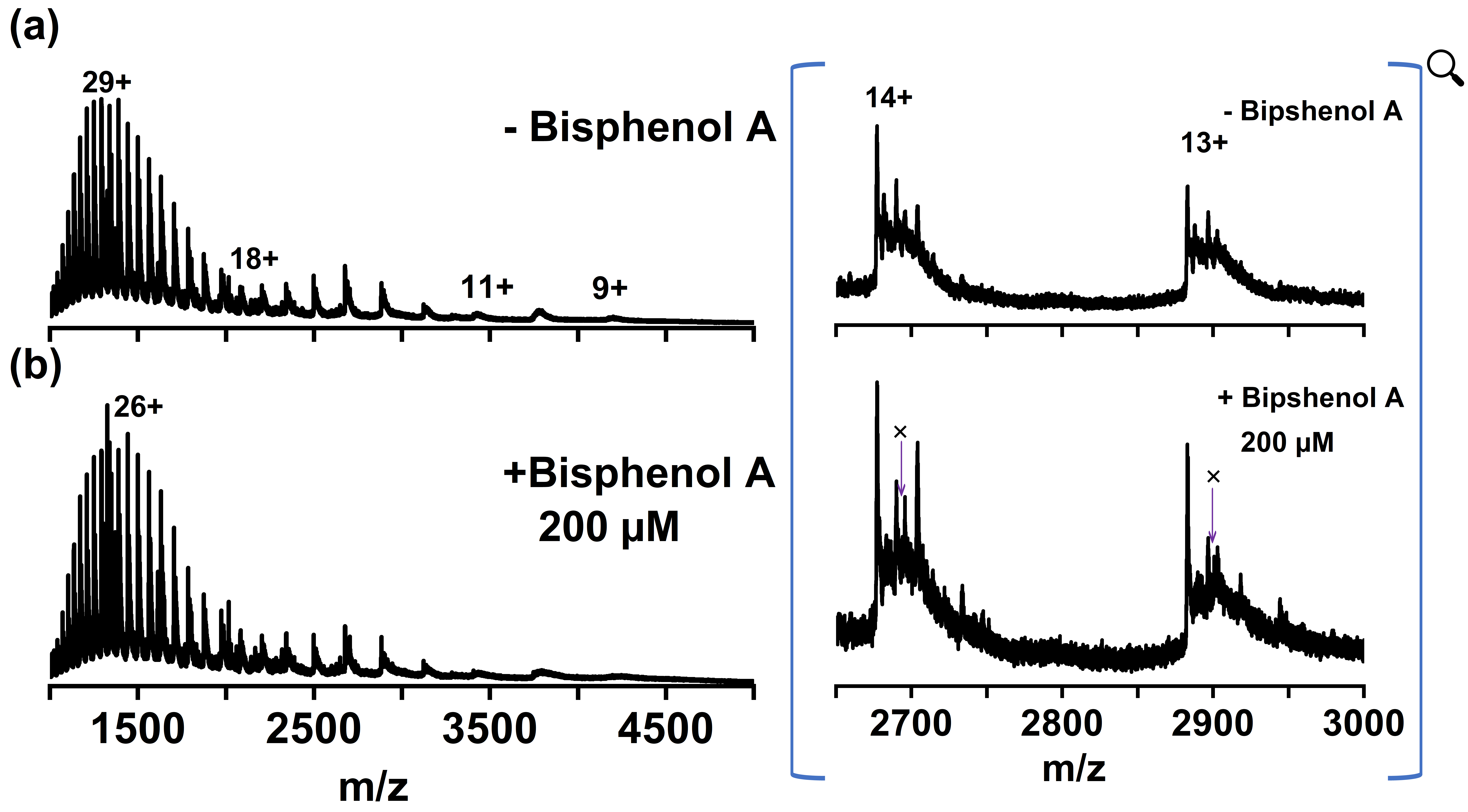
**

**Figure SI1. nMS of AR-AF1 (4 µM) analysed from AmAc (55 mM) pH 6.8 in the absence (a) and presence (b) of Bisphenol A at 200 µM. Each spectrum is enlarged in the region of 2650 to 3000 m/z, and arrows indicate the expected m/z of the hypothetical AR-AF1-BPA complex, which is not observed. Additional adducts are from plasticiser contaminants, all of which are present in both spectra. Signal intensity is lower in (b), likely due to competing ionisation from unbound BPA. Molecular weight values of AR-AF1 are shown in Table SI1.**

**
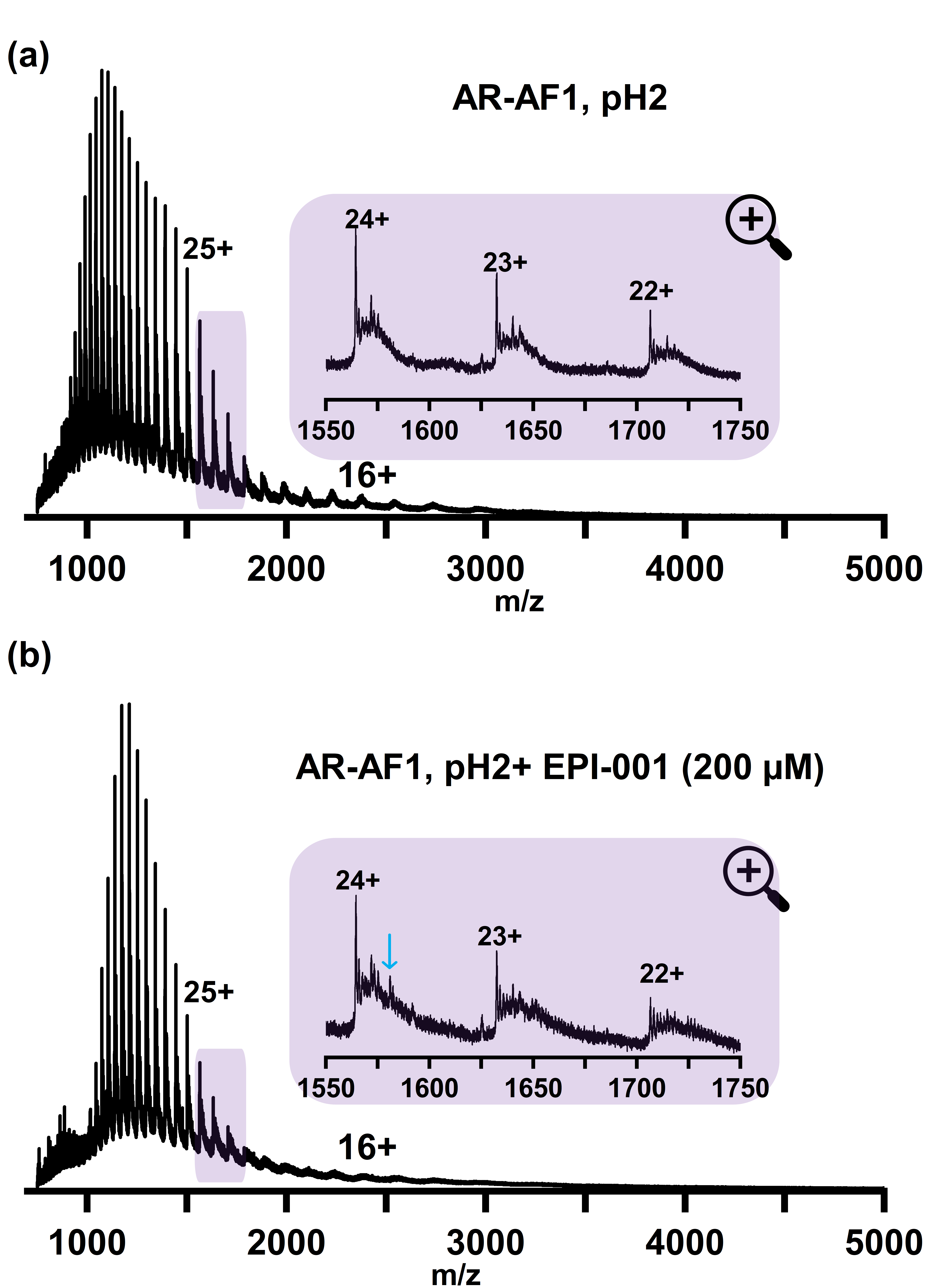
**

**Figure SI2. nMS of AR-AF1 (4 µM) in AmAc (10 mM) at pH 2 in the absence (a) and presence (b) of EPI-001 (200 µM, 1% DMSO). Insets show enlarged region of 22+ to 24+ charge states as the blue arrow shows trace amount of complex with EPI-001 under these conditions.**

**
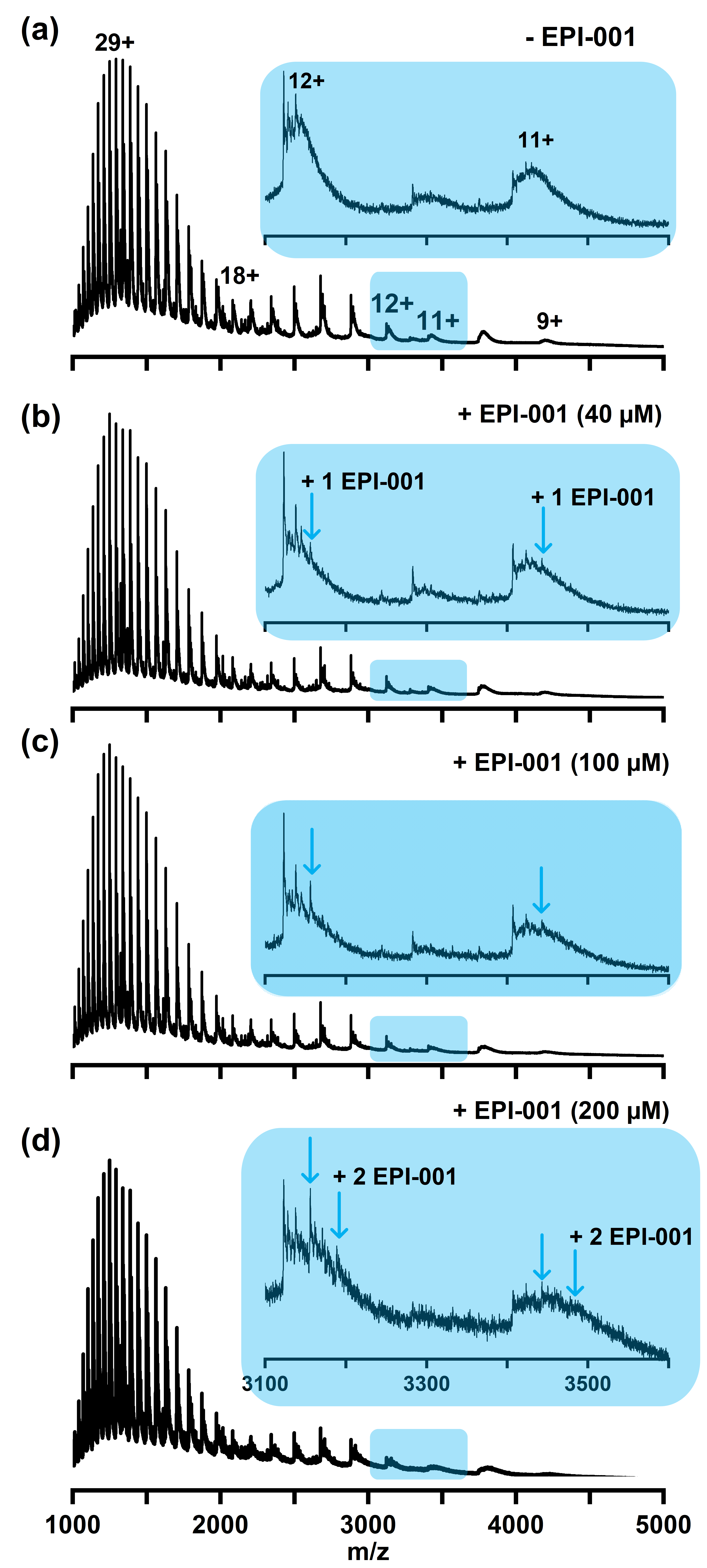
**

**Figure SI3. nMS of AR-AF1 (4 µM) analysed from AmAc (55 mM), 1% DMSO in the absence (a) and presence of EPI-001 at 40 µM (b), 100 µM (c) and 200 µM (d). Each spectrum is enlarged in the region of 3100 to 3600 m/z, where one (blue arrow) and two molecules (2 blue arrows) of EPI-001 binding to charge states 11+ and 12 is shown, additional adducts are from plasticiser contaminants.**

**

**

**Figure SI4. Full spectra corresponding to the data shown in Figure 3 of the manuscript. nMS of AR-TAU5 (a, b) and AR-TAU1 (c, d) in the absence (a, c) and presence (b, d) of EPI-001.**





**Figure SI5. nMS of AR-TAU5 (a, b) and AR-TAU1 (c, d) ionised from starting solutions of 10 μM protein concentration, 55 mM AmAc pH 6.8, 1% DMSO in the absence (a, c) and presence (b, d) of EPI-001 at 100 µM. Insets show enlarged region of 7+ and 8+ charge states to show complexation with EPI-001, using red arrow (AR-TAU5) or colour coded red within the spectrum (AR-TAU1) , additional adducts are from plasticiser contaminants. Molecular weight values of AR-TAU1 and AR-TAU5 are shown in Table SI1.**





**Figure SI6. nMS of AR-TAU5 (a, b) and AR-TAU1 (c, d) ionised from starting solutions of 10 μM protein concentration, 55 mM AmAc pH 6.8, 1% DMSO in the absence (a, c) and presence (b, d) of EPI-001 at 200 µM. Insets show enlarged region of 7+ and 8+ charge states to show complexation with EPI-001, using red arrow (TAU5) or colour coded (TAU1) red and blue for 1 or 2 bound EPI-001.**


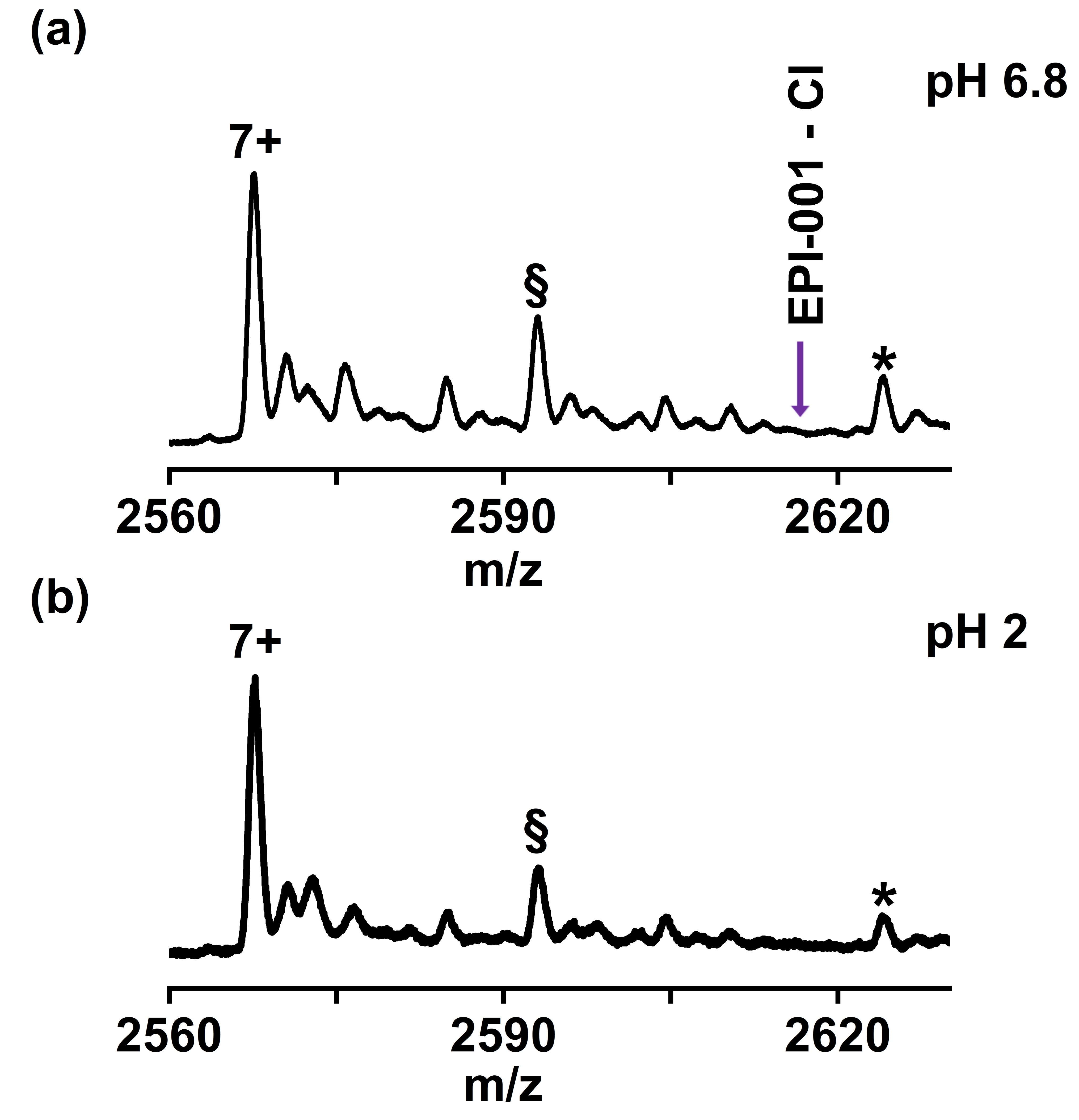


**Figure SI7. nMS of AR-TAU5 (10 μM) incubated in Tris buffer + EPI-001 (200 µM) for 24h and subsequently buffer exchanged into 55 mM AmAc pH 6.8 (a). The sample was then incubated with formic acid for 20 min for analysis at pH2 (b). * indicates the 1:1 AR-TAU5-EPI-001 complex, and § indicates the bound plasticiser molecule.**


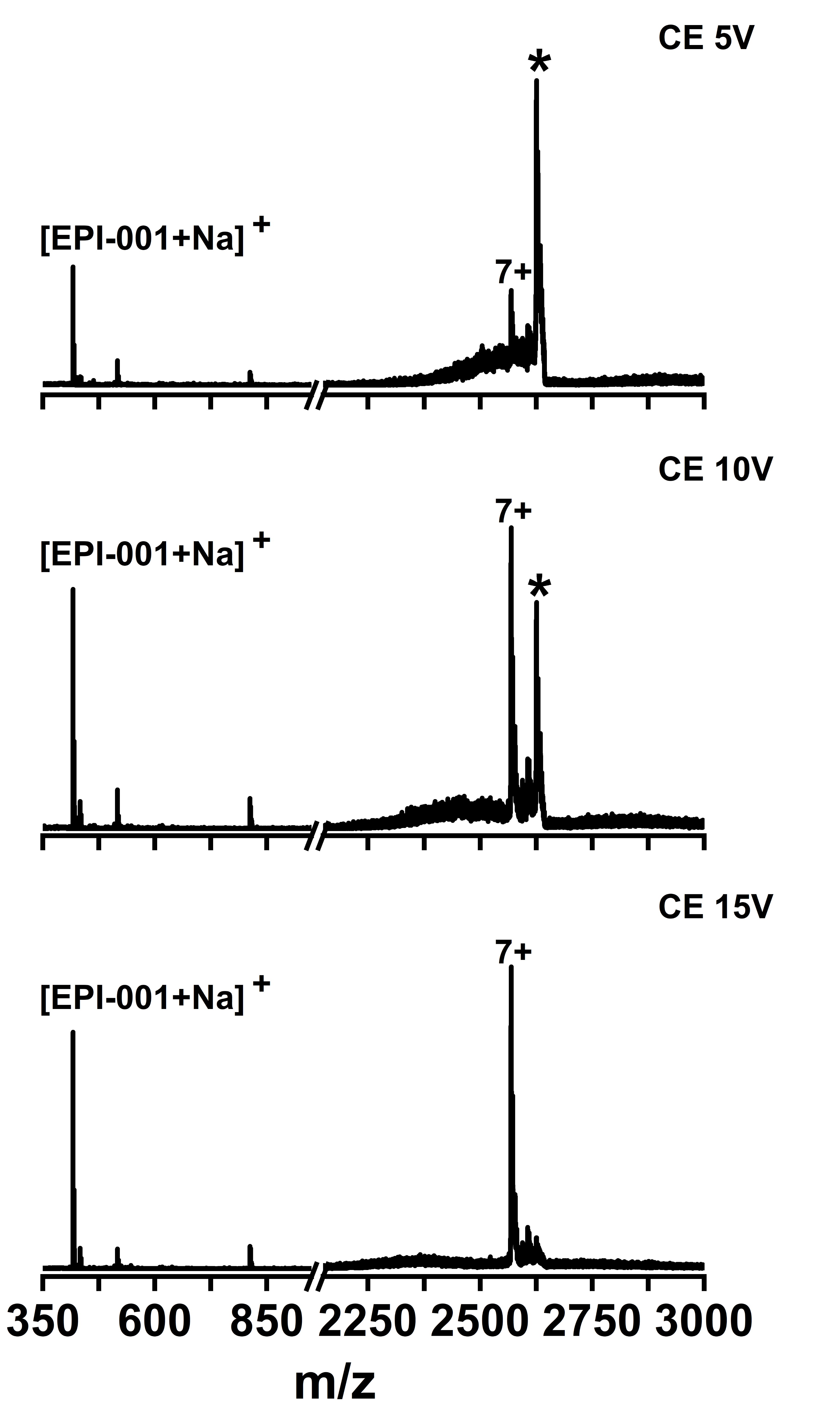


**Figure SI8. MS/MS experiments of the isolated 7+ charge state of the 1:1 AR-TAU5:EPI-001 complex ionised from starting solutions of 10 μM protein concentration, 55 mM AmAc pH 6.8, in the presence of EPI-001 at 200 µM at multiple collisional energies (CE 5 -15 V). Complexation with EPI-001 is indicated by the symbol * for 1:1 protein to drug complex, whereas the symbol // indicates an X- axis break between 950-2150 m/z.**

**
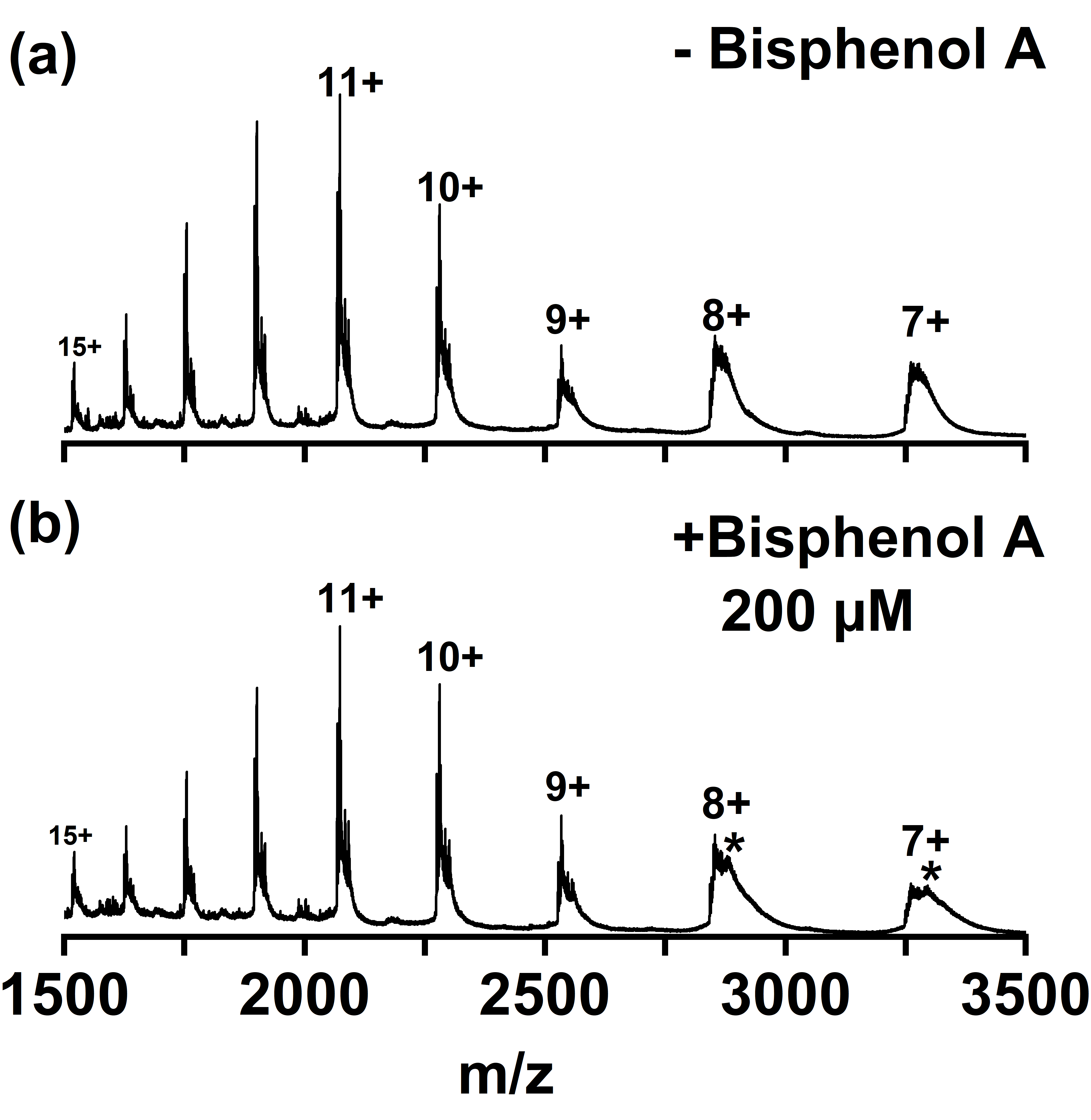
**

**Figure SI9. nMS of AR-TAU1 (10 µM) analysed from AmAc (55 mM) pH 6.8 in the absence (a) and presence (b) of Bisphenol A at 200 µM. A low amount of binding is seen in the 8+ and 7+ charge states as indicted by *.**

**
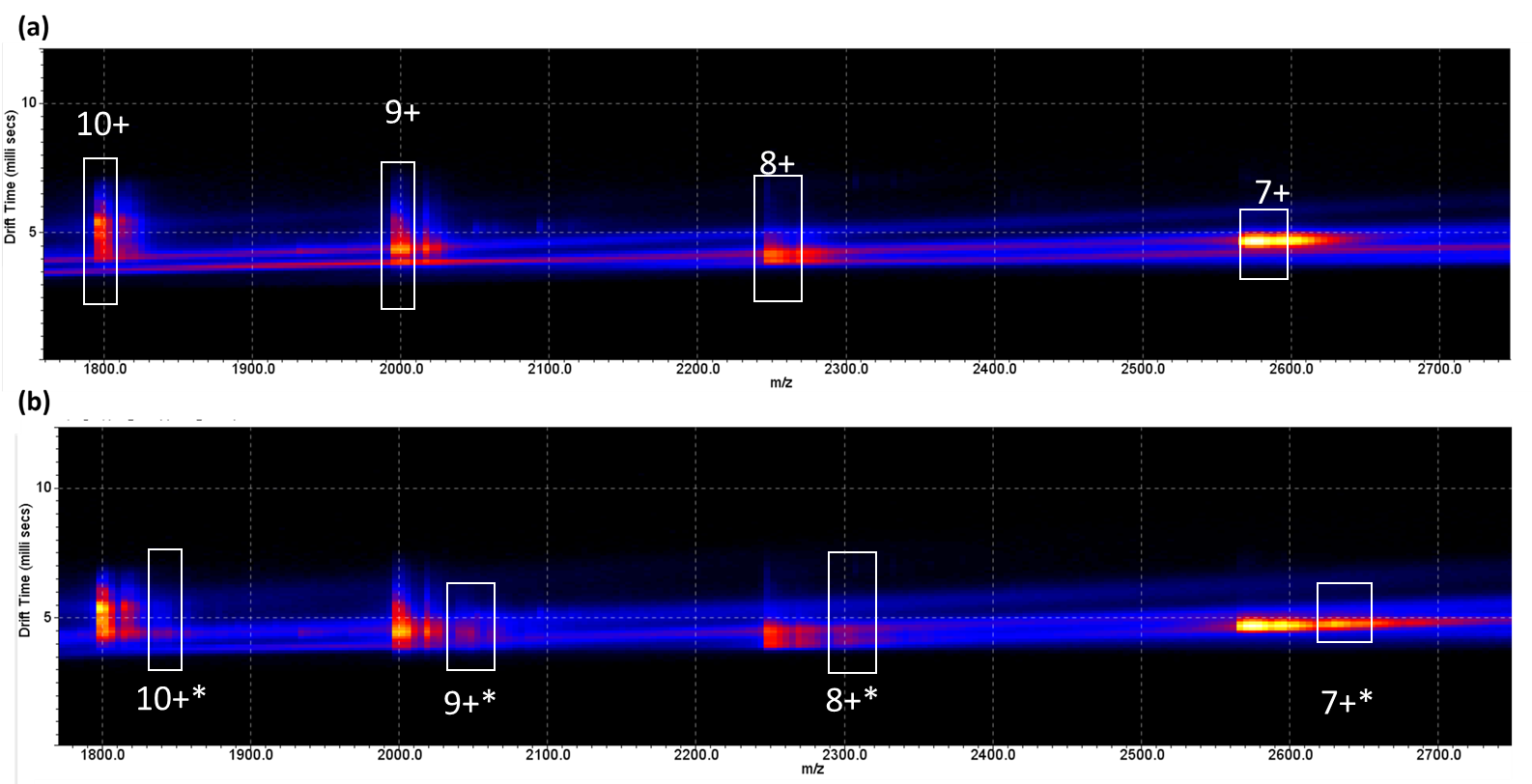
**

**Figure SI10. Representative 2D heatmaps showing some background interference of the IMMS data. (a) AR-TAU5 (b) AR-TAU5 + EPI-001.**

**
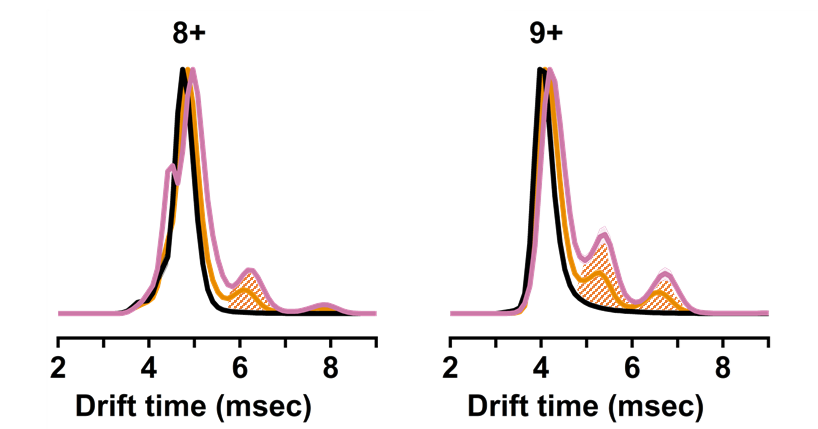
**

**Figure SI11. Black line shows ATD of AR-TAU1 in charge states 8+ and 9+ in the absence of EPI-001. Orange line shows ATD of AR-TAU1:EPI-001 in a 1:1 stoichiometry, and the pink line shows the ATD of AR-TAU1:EPI-001 in a 1:2 stoichiometry. The signal at higher arrival times is artificially higher due to lower signal intensity for the main peak. Shaded regions show signal interference.**


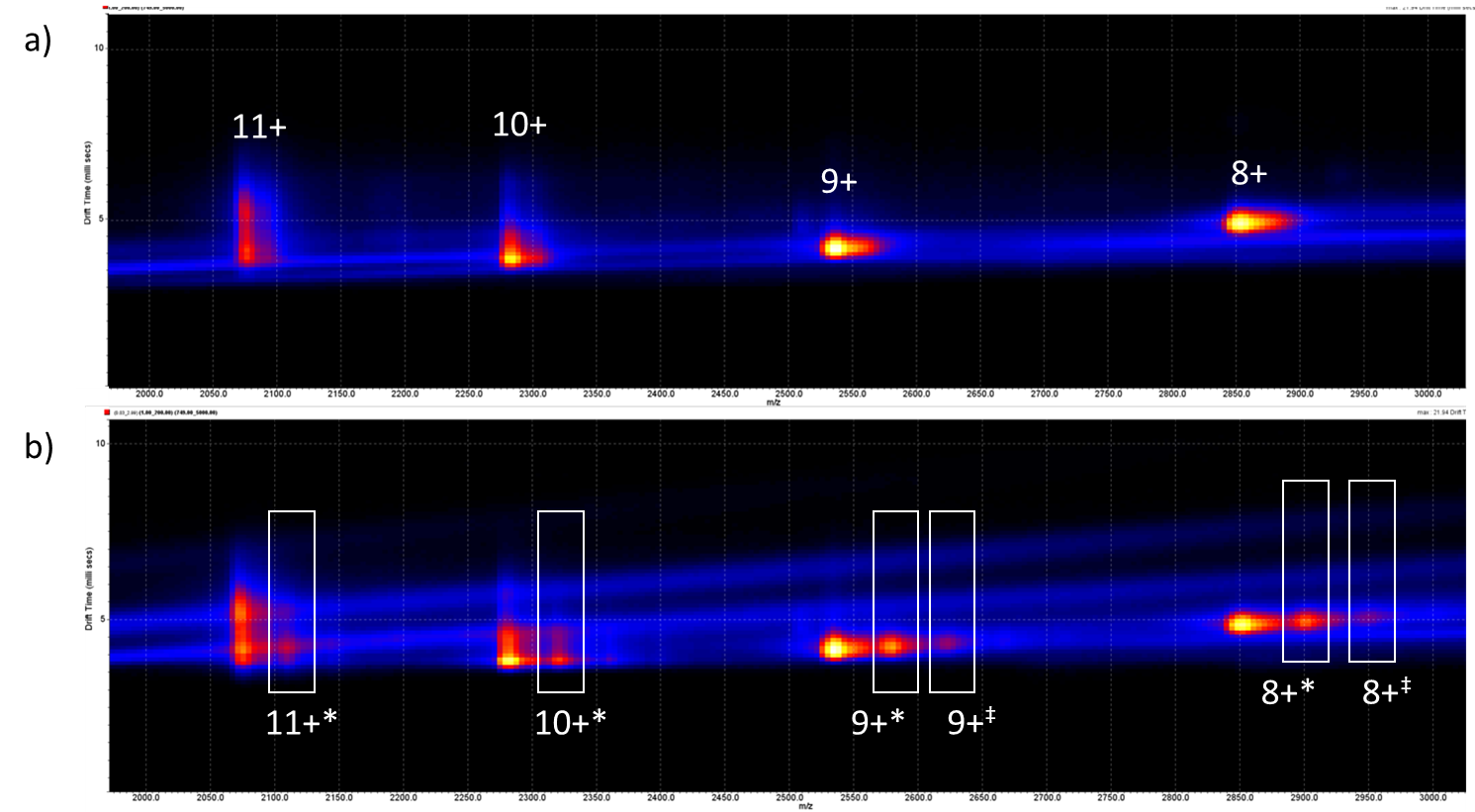


**Figure SI12. Representative 2D heatmaps showing some background interference of the IMMS data. (a) AR-TAU1 (b) AR-TAU1 + EPI-001.**


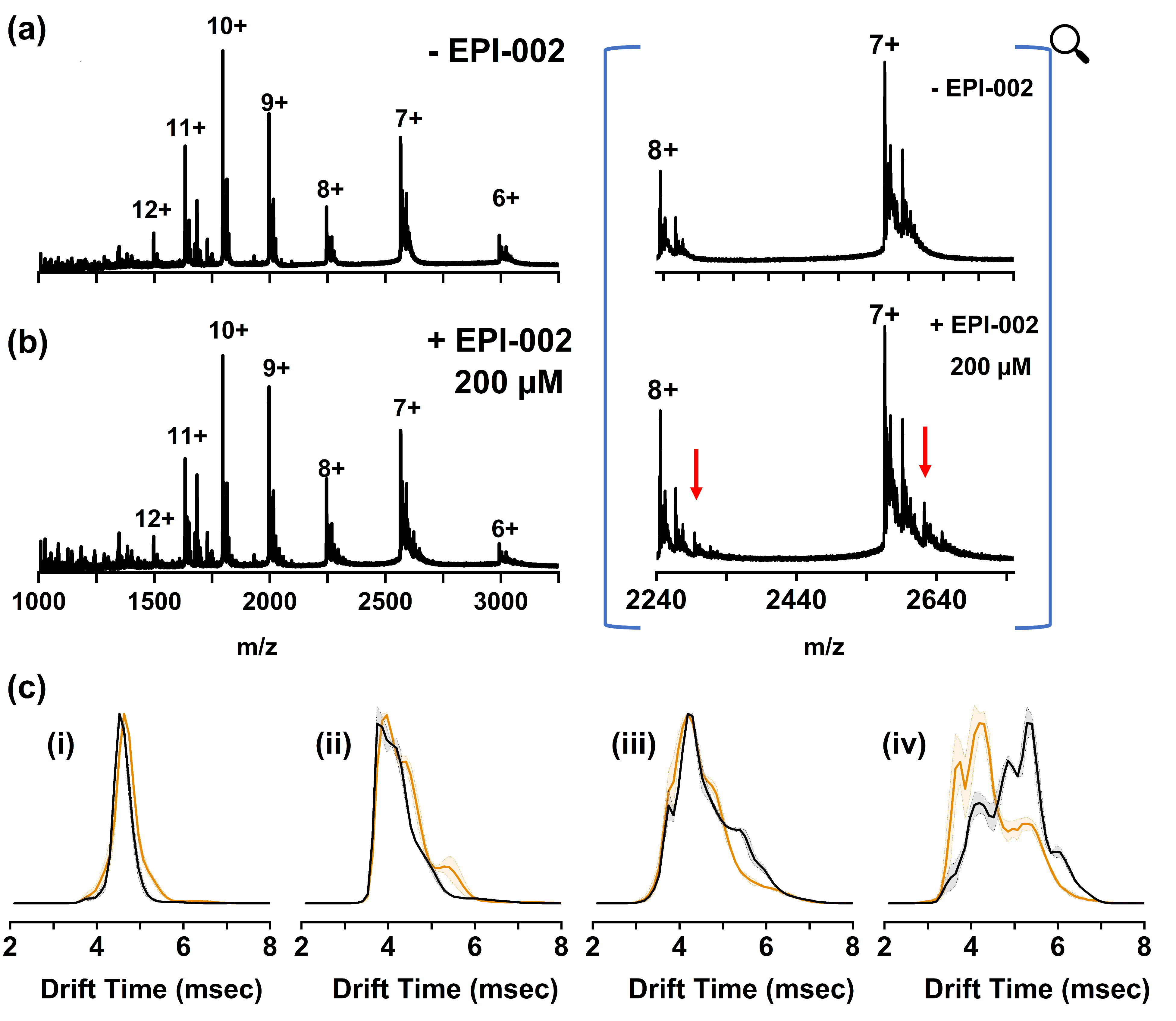


**Figure SI13. (a) nMS of AR-TAU5 (10 μM) ionised from 55 mM AmAc 1% DMSO. (b) As a, with the addition of EPI-002 to the starting solution (200 μM). (c) IM traces of charge states 7+ (i), 8+ (ii), 9+ (iii) and 10+ (iv) of AR-TAU5. Black line: control (1% DMSO). Orange line: protein-drug complex at 1:1 stoichiometry when analysed from solution containing 200 µM EPI-002. The solid lines correspond to the average of 3 repeats across different days, and the shaded area corresponds to standard deviation.**

**Table SI1. Theoretical and measured mass of AR-AF1, AR-TAU1, and AR-TAU5.**

| **Protein** | **Theoretical molecular weight from sequence/ Da** | **Measured molecular weight/ Da** |
| --- | --- | --- |
| **AR-AF1** | 38 044 | 37 473 ± 18 |
| **AR- TAU1** | 22 856 | 22 722.09 ± 5 |
| **AR-TAU5** | 18 812 | 17 956 ± 5 |

**Table SI2.** The sequences of all AR protein constructs.

| **Protein** | **Sequence** |
| --- | --- |
| **AR-AF1** | *MGHHHHHHHHHHSSGHIDDDDKHMASSGSS –*  LPQQLPAPPDEDDSAAPSTLSLLGPTFPGLSSCSADLKDILSEASTMQLLQQQQQEAVSEGSSSGRAREASGAPTSSKDNYLGGTSTISDNAKELCKAVSVSMGLGVEALEHLSPGEQLRGDCMYAPLLGVPPAVRPTPCAPLAECKGSLLDDSAGKSTEDTAEYSPFKGGYTKGLEGESLGCSGSAAAGSSGTLELPSTLSLYKSGALDEAAAYQSRDYYNFPLALAGPPPPPPPPHPHARIKLENPLDYGSAWAAAAAQCRYGDLASLHGAGAAGPGSGSPSAAASSSWHTLFTAEEGQLYGPC**GGGGGGGGGGGGGGGGGGGGGGGG**EAGAVAPYGYTRPPQGLAGQES |
| **AR-AF1-TAU1** | *MGHHHHHHHHHHSSGHIDDDDKHMASSGSS –*  LPQQLPAPPDEDDSAAPSTLSLLGPTFPGLSSCSADLKDILSEASTMQLLQQQQQEAVSEGSSSGRAREASGAPTSSKDNYLGGTSTISDNAKELCKAVSVSMGLGVEALEHLSPGEQLRGDCMYAPLLGVPPAVRPTPCAPLAECKGSLLDDSAGKSTEDTAEYSPFKGGYTKGLEGESLGCSGSAAAGSSG |
| **AR-AF1-TAU5** | *MGHHHHHHHHHHSSGHIDDDDKHMASSGSS –*  SSGTLELPSTLSLYKSGALDEAAAYQSRDYYNFPLALAGPPPPPPPPHPHARIKLENPLDYGSAWAAAAAQCRYGDLASLHGAGAAGPGSGSPSAAASSSWHTLFTAEEGQLYGPC**GGGGGGGGGGGGGGGGGGGGGGGG**EAGAVAPYGYTRPPQGLAGQES |

**Table SI3.** Solution conditions of AR constructs used in the study.

| **Protein construct** | **Buffer exchange** | **Final protein concentration** | **Final AmAc concentration** | **Final small molecule concentration** |
| --- | --- | --- | --- | --- |
| **AR-AF1** | Dialysis into 10 mM AmAC | 4 µM | 10 or 55 mM | 40, 100 or 200 µM |
| **AR- TAU5** | Dialysis into 10 mM AmAC | 10 µM | 55 mM | 100 or 200 µM |
| **AR-TAU1** | Dialysis into 55 mM AmAC | 10 µM | 55 mM | 100 or 200 µM |

**Appendix I: EPI-002 synthesis**

All reagents and starting materials were obtained from commercial sources and used as received without further purification, unless otherwise stated. Acetone, anhydrous *N*,*N*-dimethylformamide, ethanol, diethyl ether, ethyl acetate and petroleum ether (40–60 °C) were used as obtained from suppliers without further purification. Anhydrous dichloromethane and toluene were purified using a PureSolv SPS-400-5 Solvent Purification System. All reactions were performed using round-bottom flasks or microwave vials of appropriate volume. Reactions were carried out at elevated temperatures using a temperature regulated hotplate/stirrer and DrySyn block with a contact thermometer. Room temperature generally refers to ~ 20 °C. Reactions requiring a reduced temperature were performed using an ice bath (0 °C) with a temperature probe unless otherwise stated. Brine refers to a saturated aqueous solution of sodium chloride.

Reactions were monitored by thin layer chromatography (TLC) using Merck silica gel 60 covered aluminium backed plated F254. TLC plates were visualized under UV light and staining using potassium permanganate solution, vanillin or ninhydrin. Flash column chromatography was performed with Fluorochem silica gel 60 (40−63 μm).

^1^H and ^13^C NMR spectra were recorded on a Bruker DRX 500 NMR spectrometer 500 and 126 MHz, respectively or on a Bruker AV3 400 NMR spectrometer at 400 and 101 MHz using the deuterated solvent as the internal deuterium lock. Chemical shifts (δ) are reported in ppm relative to the residual protic solvent where δ (CDCl_3_) = 7.26 ppm (^1^H) and δ (CDCl_3_) = 77.16 ppm. ^1^H signals are described as singlets (s), doublets (d), triplets (t), quartets (q), quintets (quin.), multiplets (m), broad (br), app (apparent) or a combination of these and coupling constants are measured in Hz.

Low-resolution mass spectra were obtained using an Agilent Technologies 1200 series instrument with a 6130 single quadrupole LC/MS using a poroshell EC-C18 column. Analysis was performed using a gradient method, eluting with 5–95% acetonitrile (containing 5nM ammonium acetate)/water (containing 5nM ammonium acetate) over 18 minutes at a flow rate of 1 mL/min, with UV monitoring at 254 nm.

Chiral HPLC data were obtained on an Agilent 1200 series HPLC using Daicel chiralcel OD-H column using an isocratic method (hexane:isopropanol:ethanol, 50:25:25) over 40 minutes at a flow rate of 1 mL/min and UV monitoring at 254 nm.

**Synthetic route towards EPI-002**

**(*R*)-(2,2-Dimethyl-1,3-dioxolan-4-yl)methyl 4ʹ-methylbenzenesulfonate**^1^

An oven-dried flask was flushed with nitrogen and charged with anhydrous dichloromethane (30 mL) then cooled to 0 °C. (*S*)-(2,2-Dimethyl-1,3-dioxolan-4-yl)methanol (1.87 mL, 15.1 mmol) was added followed by sequential addition of triethylamine (2.50 mL, 18.2 mmol), *p*-toluenesulfonyl chloride (3.46 g, 18.2 mmol) and 4-dimethylaminopyridine (0.185 g, 1.51 mmol). The reaction mixture was warmed to room temperature and stirred for 16 h. The reaction mixture was diluted with dichloromethane and washed with water (30 mL) and brine (30 mL), dried (MgSO_4_), filtered and concentrated *in vacuo.* This afforded (*R*)-(2,2-dimethyl-1,3-dioxolan-4-yl)methyl 4-methylbenzenesulfonate (4.19 g, 97%) as a yellow oil. Spectroscopic data were consistent with the literature^1^. ^1^H NMR (500 MHz, CDCl_3_) δ 7.79 (d, *J* = 8.2 Hz, 2H), 7.35 (d, *J* = 8.2 Hz, 2H), 4.27 (quin., *J* = 5.7 Hz, 1H), 4.07–4.00 (m, 2H), 3.97 (dd, *J* = 10.1, 6.2 Hz, 1H), 3.76 (dd, *J* = 8.8, 5.2 Hz, 1H), 2.45 (s, 3H), 1.34 (s, 3H), 1.31 (s, 3H); ^13^C NMR (126 MHz, CDCl_3_) δ 145.2, 132.8, 130.0 (2 × CH), 128.1 (2 × CH), 110.2, 73.0, 69.6, 66.3, 26.8, 25.3, 21.8; LCMS (ESI) m/z: [M + H]^+^ Calcd. for C_13_H_19_O_5_S 287.1; Found 287.3 at 8.09 mins.

**(*S*)-4-(2-(4-((2,2-Dimethyl-1,3-dioxolan-4-yl)methoxy)phenyl)propan-2-yl)phenol**^2^

Bisphenol A (6.67 g, 29.2 mmol) was dissolved in anhydrous *N,N*-dimethylformamide (25 mL) and sodium hydride (1.17 g, 29.2 mmol; 60% in mineral oil) was added slowly at room temperature. The mixture was stirred for 20 minutes under nitrogen before (*R*)-(2,2-dimethyl-1,3-dioxolan-4-yl)methyl 4-methylbenzenesulfonate (4.19 g, 14.6 mmol) in *N,N*-dimethylformamide (5 mL) was slowly added. The reaction mixture was stirred at 60 °C for 20 h. The mixture was extracted with ethyl acetate (3 × 80 mL) and the combined organic extracts were washed with water (4 × 50 mL), brine (50 mL), dried (MgSO_4_), filtered and concentrated *in vacuo*. Purification by flash column chromatography (gradient elution, 0–15% diethyl ether in petroleum ether) afforded (*S*)-4-(2-(4-((2,2-dimethyl-1,3-dioxolan-4-yl)methoxy)phenyl)propan-2-yl)phenol (2.74 g, 55%) as a yellow oil. Spectroscopic data were consistent with the literature^2^. ^1^H NMR (500 MHz, CDCl_3_) δ 7.16–7.10 (m, 2H), 7.08 (d, *J* = 8.7 Hz, 2H), 6.85–6.77 (m, 2H), 6.76–6.69 (m, 2H), 4.91 (s, 1H), 4.47 (app quin., *J* = 6.0 Hz, 1H), 4.17 (app dd, *J* = 8.5, 6.0 Hz, 1H), 4.04 (app dd, *J* = 9.5, 6.0 Hz, 1H), 3.97–3.84 (m, 2H), 1.63 (s, 6H), 1.47 (s, 3H), 1.41 (s, 3H); ^13^C NMR (126 MHz, CDCl_3_) δ 156.5, 153.5, 143.8, 143.4, 128.1 (2 × CH), 127.9 (2 × CH), 114.9 (2 × CH), 114.0 (2 × CH), 109.9, 74.2, 68.9, 67.1, 41.8, 31.2 (2 × CH_3_), 26.9, 25.5; LCMS (ESI) m/z: [M − H]^+^ Calcd. for C_21_H_25_O_4_ 341.2; Found 341.2 at 8.78 mins.

**(*R*)-3-(4-(2-(4-(((*S*)-2,2-Dimethyl-1,3-dioxolan-4-yl)methoxy)phenyl)propan-2-yl)phenoxy)propane-1,2-diol^4^**

(*S*)-4-(2-(4-((2,2-Dimethyl-1,3-dioxolan-4-yl)methoxy)phenyl)propan-2-yl)phenol (0.500 g, 1.46 mmol) was dissolved in ethanol (5 mL) and (*R*)-oxiran-2-ylmethanol (0.116 mL, 1.75 mmol) and triethylamine (0.204 mL, 1.46 mmol) were added. The reaction mixture was stirred at 85 °C for 16 h in a sealed tube. After cooling to room temperature, the reaction mixture was concentrated *in vacuo*. Purification by flash column chromatography (gradient elution, 60–80% ethyl acetate in petroleum ether) afforded (*R*)-3-(4-(2-(4-(((*S*)-2,2-dimethyl-1,3-dioxolan-4-yl)methoxy)phenyl)propan-2-yl)phenoxy)propane-1,2-diol (0.340 g, 56%) as a colourless oil. Spectroscopic data were consistent with the literature.^4^ ^1^H NMR (500 MHz, CDCl_3_) δ 7.16–7.09 (m, 4H), 6.81 (d, *J* = 8.5 Hz, 4H), 4.46 (quin., *J* = 5.9 Hz, 1H), 4.16 (dd, *J* = 8.2, 6.6 Hz, 1H), 4.09 (quin., *J* = 5.4 Hz, 1H), 4.07–3.99 (m, 3H), 3.94–3.87 (m, 2H), 3.83 (dd, *J* = 11.4, 3.8 Hz, 1H), 3.74 (dd, *J* = 11.4, 5.4 Hz, 1H), 1.63 (s, 6H), 1.45 (s, 3H), 1.40 (s, 3H); ^13^C NMR (126 MHz, CDCl_3_) δ 156.6, 156.4, 144.0, 143.6, 128.0 (2 × CH), 127.9 (2 × CH), 114.1 (4 × CH) 109.9, 74.2, 70.5, 69.3, 69.0, 67.1, 63.9, 41.9, 31.2 (2 × CH_3_), 26.9, 25.5; LCMS (ESI) m/z: [M + Na]^+^ Calcd. for C_24_H_32_NaO_6_ 439.2; Found 439.3 at 8.23 mins.

**(*S*)-3-(4-(2-(4-(((*S*)-2,2-Dimethyl-1,3-dioxolan-4-yl)methoxy)phenyl)propan-2-yl)phenoxy)-2-hydroxypropyl 4-methylbenzenesulfonate**

(*R*)-3-(4-(2-(4-(((*S*)-2,2-Dimethyl-1,3-dioxolan-4-yl)methoxy)phenyl)propan-2-yl)phenoxy)propane-1,2-diol (0.313 g, 0.751 mmol) was dissolved in anhydrous toluene (10 mL) and dibutyltin(IV) oxide (0.00370 g, 0.0150 mmol) was added and the resulting mixture was stirred for 1 h at room temperature. *N*,*N*-Diisopropylethylamine (0.157 mL, 0.901 mmol) was added, the mixture was stirred for 5 minutes and then *p*-toluenesulfonyl chloride (0.151 g, 0.789 mmol) was added slowly. The reaction mixture was stirred for 18 h at room temperature and then quenched by the addition of 1 m aqueous hydrochloric acid (10 mL). The mixture was extracted with dichloromethane (4 × 30 mL) and the combined organic extracts were washed with brine (100 mL), dried (MgSO_4_), filtered and concentrated *in vacuo.* Purification by flash column chromatography (gradient elution, 20–30% ethyl acetate in petroleum ether) afforded (*S*)-3-(4-(2-(4-(((*S*)-2,2-dimethyl-1,3-dioxolan-4-yl)methoxy)phenyl)propan-2-yl)phenoxy)-2-hydroxypropyl 4-methylbenzenesulfonate (0.344 g, 80%) as a colourless oil. ^1^H NMR (500 MHz, CDCl_3_) δ 7.83–7.76 (m, 2H), 7.31 (d, *J* = 8.0 Hz, 2H), 7.17–7.08 (m, 4H), 6.86–6.78 (m, 2H), 6.76–6.68 (m, 2H), 4.46 (quin., *J* = 6.0 Hz, 1H), 4.25–4.13 (m, 4H), 4.04 (dd, *J* = 9.5, 5.4 Hz, 1H), 3.96 (d, *J* = 4.4 Hz, 2H), 3.90 (td, *J* = 9.1, 6.0 Hz, 2H), 2.42 (s, 3H), 1.63 (s, 6H), 1.46 (s, 3H), 1.40 (s, 3H); ^13^C NMR (126 MHz, CDCl_3_) δ 156.6, 156.0, 145.3, 144.2, 143.6, 132.6, 130.1 (2 × CH), 128.2 (2 × CH), 127.9 (2 × CH), 127.9 (2 × CH), 114.1 (2 × CH), 114.0 (2 × CH), 109.9, 74.2, 70.4, 69.0, 68.2, 67.9, 67.1, 41.9, 31.2 (2 × CH_3_), 26.9, 25.5, 21.8; LCMS (ESI) m/z: [M + NH_4_]^+^ Calcd. for C_31_H_42_NO_8_S 588.2; Found 588.3 at 9.64 mins.

**(*S*)-1-Chloro-3-(4-(2-(4-(((*S*)-2,2-dimethyl-1,3-dioxolan-4-yl)methoxy)phenyl)propan-2-yl)phenoxy)propan-2-ol**

(*S*)-3-(4-(2-(4-(((*S*)-2,2-Dimethyl-1,3-dioxolan-4-yl)methoxy)phenyl)propan-2-yl)phenoxy)-2-hydroxypropyl 4-methylbenzenesulfonate (0.352 g, 0.617 mmol) was dissolved in *N*,*N*-dimethylformamide (3 mL) and lithium chloride (0.392 g, 9.25 mmol) was added. The reaction mixture was stirred at 80 °C for 2 h, cooled to room temperature and quenched with water (10 mL). The mixture was extracted with ethyl acetate (4 × 30 mL) and the combined organic extracts were washed with water (5 × 20 mL), brine (20 mL), dried (MgSO_4_), filtered and concentrated *in vacuo*. This afforded (*S*)-1-chloro-3-(4-(2-(4-(((*S*)-2,2-dimethyl-1,3-dioxolan-4-yl)methoxy)phenyl)propan-2-yl)phenoxy)propan-2-ol (0.192 g, 72%) as a colourless oil. ^1^H NMR (400 MHz, CDCl_3_) δ 7.19–7.09 (m, 4H), 6.81 (dd, *J* = 8.9, 0.9 Hz, 4H), 4.46 (quin., *J* = 6.0 Hz, 1H), 4.23–4.00 (m, 5H), 3.95–3.86 (m, 2H), 3.78 (dd, *J* = 11.3, 5.3 Hz, 1H), 3.71 (dd, *J* = 11.2, 5.7 Hz, 1H), 1.63 (s, 6H), 1.46 (s, 3H), 1.40 (s, 3H); ^13^C NMR (101 MHz, CDCl_3_) δ 156.6, 156.2, 144.1, 143.6, 128.0 (2 × CH), 127.9 (2 × CH), 114.1 (2 × CH), 114.1 (2 × CH), 109.9, 74.2, 70.1, 68.9, 68.6, 67.1, 46.1, 41.9, 31.2 (2 × CH_3_), 26.9, 25.5; LCMS (ESI) m/z: [M + Na]^+^ Calcd. for C_24_H_31_ClNaO_5_ 457.2; Found 457.3 at 9.33 mins.

**(*R*)-3-(4-(2-(4-((*S*)-3-Chloro-2-hydroxypropoxy)phenyl)propan-2-yl)phenoxy)propane-1,2-diol (EPI-002)^4^**

(*S*)-1-Chloro-3-(4-(2-(4-(((*S*)-2,2-dimethyl-1,3-dioxolan-4-yl)methoxy)phenyl)propan-2-yl)phenoxy)propan-2-ol (0.192 g, 0.442 mmol) was dissolved in acetone (6 mL) and 1 m aqueous hydrochloric acid (10 mL) was added. The reaction mixture was stirred at 50 °C for 0.5 h then concentrated *in vacuo.* Purification by flash column chromatography (gradient elution, 50–100% ethyl acetate in petroleum ether) afforded (*R*)-3-(4-(2-(4-((*S*)-3-chloro-2-hydroxypropoxy)phenyl)propan-2-yl)phenoxy)propane-1,2-diol (0.141 g, 81%) as a colourless oil. Spectroscopic data were consistent with the literature.^4^ ^1^H NMR (400 MHz, CDCl_3_) δ 7.14 (d, *J* = 8.8 Hz, 4H), 6.81 (d, *J* = 8.8 Hz, 4H), 4.19 (quin., *J* = 5.4 Hz, 1H), 4.12–3.98 (m, 5H), 3.89–3.65 (m, 4H), 2.58 (d, *J* = 4.7 Hz, 1H), 2.53 (d, *J* = 6.0 Hz, 1H), 2.00 (t, *J* = 6.0 Hz, 1H), 1.63 (s, 6H); ^13^C NMR (101 MHz, CDCl_3_) δ 156.4, 156.2, 144.1, 143.9, 128.0 (4 × CH), 114.1 (4 × CH), 70.5, 70.1, 69.3, 63.9, 41.9, 31.2 (2 × CH_3_); LCMS (ESI) m/z: [M + Na]^+^ Calcd. for C_21_H_27_ClNaO_5_ 417.1; Found 417.0 at 7.52 mins, purity = 97.1%; Enantiomeric excess was determined by HPLC analysis using a chiralcel OD-H column (hexane:*^i^*PrOH:EtOH 50:25:25, flow rate 1.0 mL min^−1^); t_major_ = 31.51 min, t_minor_ = 35.75 min, er = 98.4:1.6., e.e. = 96.8%.

**References:**

(1) Xu, T.; Cuyamendous, C.; Brown, S. L.; Andreassend, S. K.; Cumming, H.; Evans, G. B.; Teesdale-Spittle, P. H.; Harvey, J. E. Gold(I)-Catalyzed, One-Pot, Oxidative Formation of 2,4-Disubstituted Thiazoles: Application to the Synthesis of a Pateamine-Related Macrodiolide. *Tetrahedron* **2021**, *88*, 132109.

(2) De Mol, E.; Fenwick, R. B.; Phang, C. T. W.; Buzón, V.; Szulc, E.; de la Fuente, A.; Escobedo, A.; García, J.; Bertoncini, C. W.; Estébanez-Perpiñá, E.; McEwan, I. J.; Riera, A.; Salvatella, X. EPI-001, A Compound Active against Castration-Resistant Prostate Cancer, Targets Transactivation Unit 5 of the Androgen Receptor. *ACS Chem. Biol.* **2016**, *11* (9), 2499–2505.

**Appendix II: Characterisation data for EPI-002**

**^1^H NMR spectra of EPI002 (400 MHz, CDCl_3_)**

**^13^C{^1^H} NMR spectra of EPI002 (101 MHz, CDCl_3_)**

**LCMS Chromatogram of EPI-002**

**
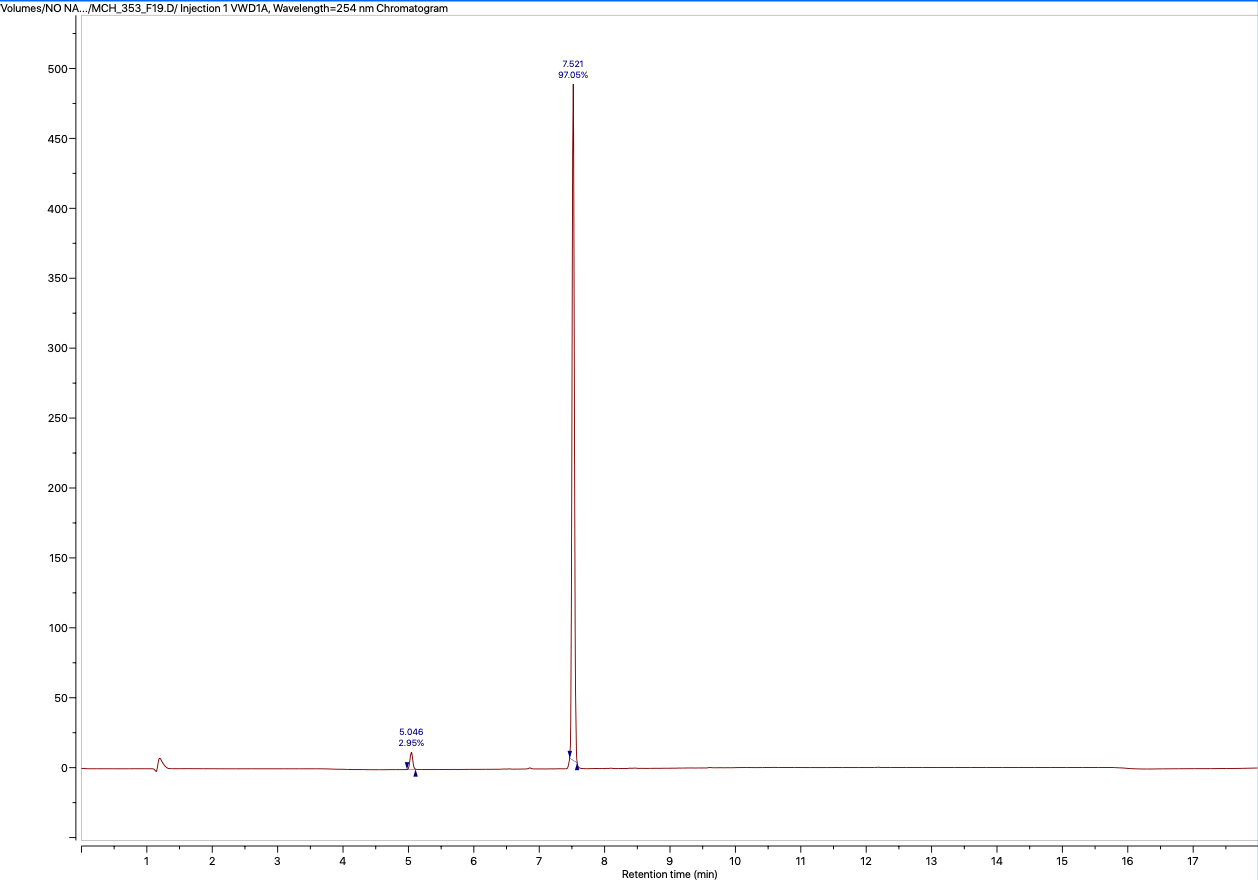
**

**Chiral HPLC traces of racemic EPI-001**

**
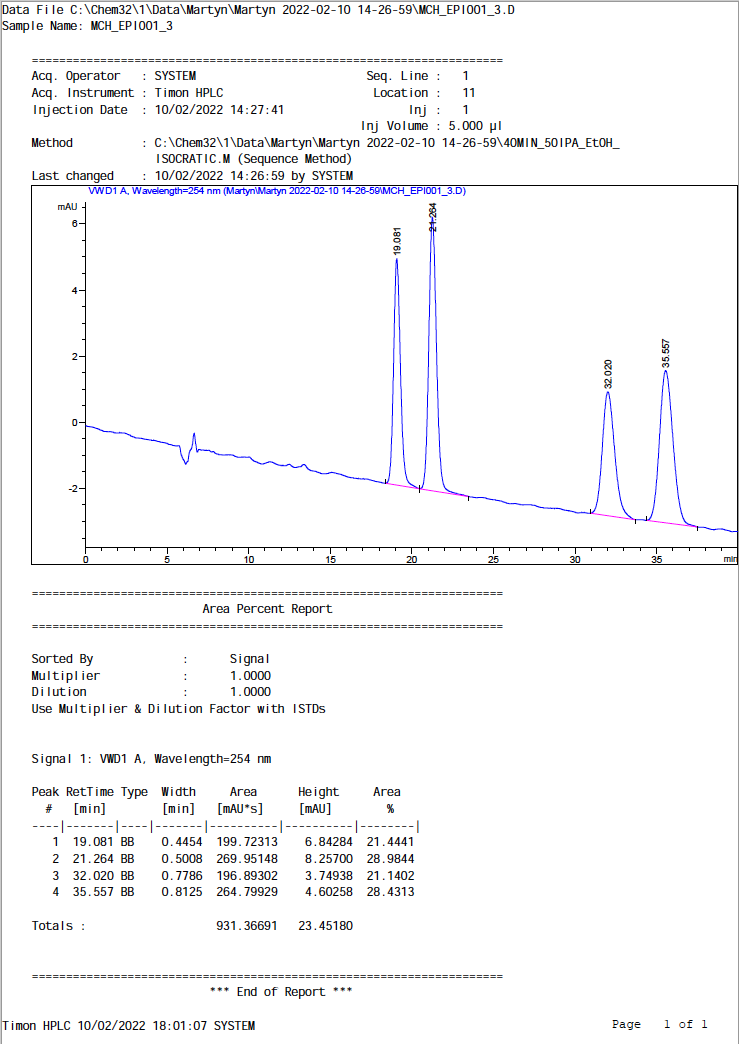
**

**Chiral HPLC trace of EPI-002**

**
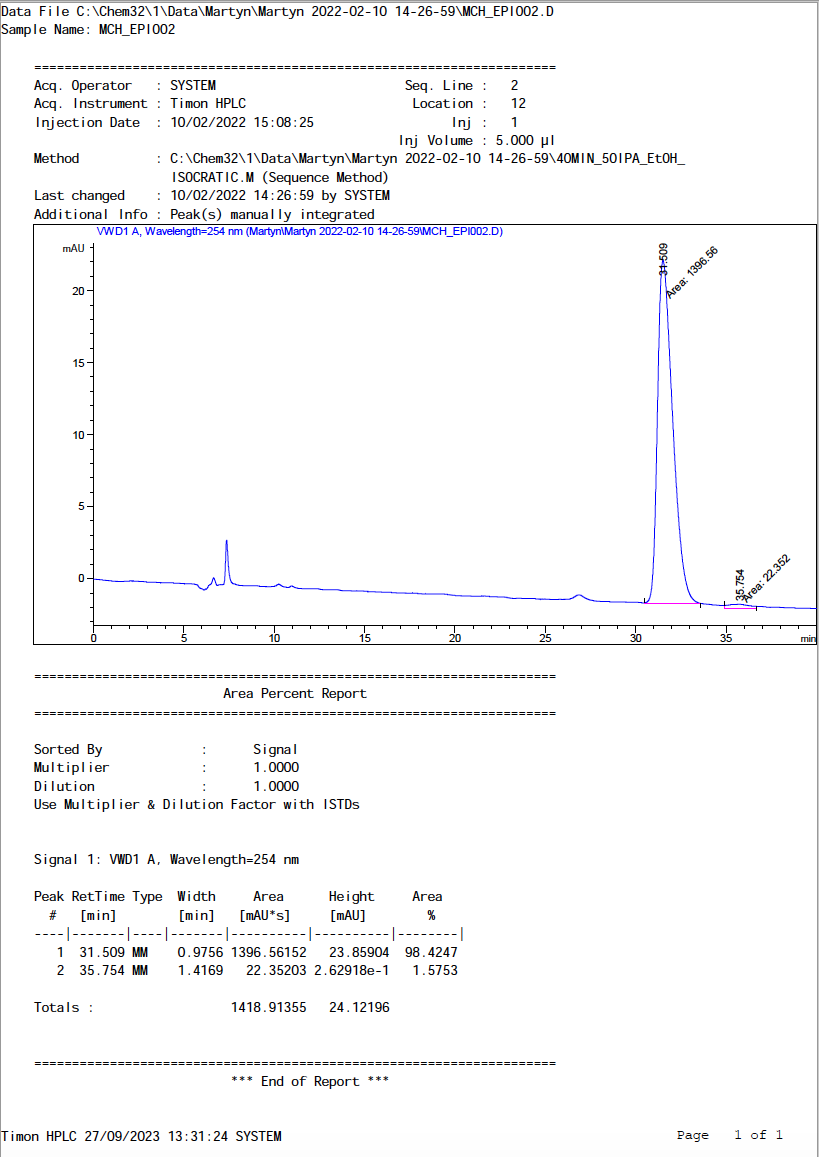
**
